# Chemical and Solvent‐Based Recycling of DGEBA‐Based Epoxy Thermoset and Carbon‐Fiber Reinforced Epoxy Composite Utilizing Imine‐Containing Secondary Amine Hardener

**DOI:** 10.1002/marc.202400678

**Published:** 2024-11-09

**Authors:** Özgün Dağlar, Tankut Türel, Christos Pantazidis, Željko Tomović

**Affiliations:** ^1^ Polymer Performance Materials Group Department of Chemical Engineering and Chemistry and Institute for Complex Molecular Systems (ICMS) Eindhoven University of Technology Eindhoven 5600 MB The Netherlands

**Keywords:** carbon‐fiber, epoxy thermoset, imine, recycling, secondary amine

## Abstract

Epoxy systems are essential in numerous industrial applications due to their exceptional mechanical properties, thermal stability, and chemical resistance. Yet, recycling epoxy networks and reinforcing materials in epoxy composites remains challenging, raising environmental concerns. The critical challenge is the recovery of well‐defined molecules upon depolymerization. To address these issues, an innovative strategy is developed utilizing imine‐containing secondary amine hardener (**M1**). The reaction of **M1** with DGEBA produced high‐performance epoxy thermoset **P1**, which exhibits Young's modulus of 2.18 GPa and tensile strength of 63.4 MPa, and excellent stability in neutral aqueous conditions. Upon carbon‐fiber reinforcement, Young's modulus and tensile strength are significantly elevated to 10.99 GPa and 328.3 MPa, respectively. The reactive secondary amine functionalities enabled the tailored network to display a well‐defined growth pattern, yielding only well‐defined molecules and intact carbon fibers upon acidic depolymerization. Consequently, the recycled polymers retained properties identical to those of **P1**. Notably, it is discovered that despite the cross‐linked nature of the epoxy networks, complete dissolution in dichloromethane facilitated straightforward solvent‐based recycling, allowing the recovery of undamaged carbon fibers and an epoxy thermoset with properties matching the virgin material. Presented novel monomer design and approach showcased two important and efficient recycling options for epoxy systems.

## Graphical abstract

The recyclability of imine‐containing epoxy thermosets, and carbon‐reinforced epoxy networks, is explored. Using an imine‐based monomer with three secondary amine functionalities reacted with DGEBA, a uniformly growing epoxy network is achieved. These high‐performance materials and composites are recycled via chemical and solvent‐based methods, including the recovery of the carbon fibers without any damage.

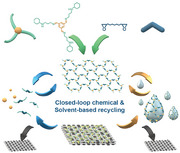

## 1 Introduction

Epoxy networks, a class of thermosetting polymers, have long been utilized in various industrial applications such as adhesives, coatings, spacecrafts, and wind turbines, owing to their exceptional mechanical properties, thermal stability, and chemical resistance.^[^ ^1^, ^2^, ^3^ ^]^ These networks are traditionally formed through the cross‐linking of epoxy monomers with curing agents (e.g., amines, anhydrides, alcohols, thiols, etc.), resulting in a robust 3D network structure.^[^ ^4^ ^]^ Among these traditional systems, the diglycidyl ether of bisphenol A (DGEBA) epoxy monomer plays a crucial role, accounting for 75% of all epoxy systems.^[^ ^5^ ^]^ The liquid nature of DGEBA and the high performance of the resulting polymers make it particularly valuable in these applications.^[^ ^4^, ^5^ ^]^ Nevertheless, despite their advantageous properties, recycling these epoxy networks, including DGEBA‐based systems, poses significant challenges due to their irreversible cross‐linking nature.^[^ ^3^, ^6^ ^]^ Traditional recycling methods, especially in systems reinforced with carbon and glass fibers utilize harsh methods, including sub‐ and supercritical conditions.^[^ ^6^, ^7^, ^8^, ^9^ ^]^ However, fibers obtained from these methods often experience a decrease in performance.^[^ ^10^, ^11^, ^12^, ^13^, ^14^ ^]^ After the removal of fibers, mixtures of various irregular oligomeric structures are obtained, posing significant challenges in separation and isolation.^[^ ^7^ ^]^ Ultimately, these materials become waste at the end of their lifespan, exacerbating environmental issues and contributing to resource depletion.^[^ ^15^, ^16^ ^]^

In light of growing environmental consciousness and the need for sustainable/recyclable materials, there is a pressing demand to develop epoxy networks with inherent recycling ability.^[^ ^3^, ^17^, ^18^, ^19^, ^20^, ^21^, ^22^, ^23^, ^24^, ^25^, ^26^ ^]^ By designing epoxy formulations that retain their superior properties while allowing for facile recycling, we can minimize the environmental impact associated with epoxy waste and promote a more circular economy.^[^ ^3^, ^17^, ^22^, ^27^ ^]^ Moreover, the development of recyclable epoxy networks holds promise for reducing dependency on fossil resources, as recycled materials can be reintroduced into the manufacturing process, reducing the need for virgin feedstocks.^[^ ^26^, ^28^, ^29^, ^30^, ^31^, ^32^ ^]^ Furthermore, for industrial applications, circular epoxy polymers must satisfy specific criteria beyond mere recyclability.^[^ ^4^, ^33^ ^]^ These criteria include the preference for liquid or low‐melting monomers, which simplifies handling and processing. Additionally, the resulting polymers should demonstrate high tensile strength, optimal Young's modulus values, elevated thermal decomposition temperatures, and hydrolytic stability.^[^ ^33^, ^34^ ^]^ These properties must remain consistent throughout the material's entire service lifespan.

In the last few decades, researchers have explored the integration of various chemically cleavable or dynamic bonds,^[^ ^3^ ^]^ such as esters,^[^ ^26^, ^35^, ^36^, ^37^ ^]^ boronic esters,^[^ ^38^ ^]^ imines,^[^ ^25^, ^28^, ^29^, ^39^, ^40^ ^]^ hydrazones,^[^ ^41^ ^]^ acetals,^[^ ^42^, ^43^, ^44^, ^45^ ^]^ disulfides,^[^ ^46^, ^47^, ^48^, ^49^, ^50^ ^]^ and hexahydro‐s‐triazines,^[^ ^51^, ^52^, ^53^ ^]^ into the structure of epoxy monomers or curing agents. While these chemistries have been effectively utilized, achieving closed‐loop recycling of epoxy networks has remained elusive and has been scarcely documented in existing literature.

The most important goal of the chemical recycling is to produce well‐defined recyclates with high yields.^[^ ^3^, ^17^, ^54^, ^55^, ^56^ ^]^ This enables the creation of new polymers with properties identical to the original, without requiring additional monomer input, thereby adhering to the principles of closed‐loop recycling. Despite the introduction of new dynamic domains, the recycling of high‐performance epoxy thermosets continues to present significant challenges.^[^ ^3^, ^17^, ^57^ ^]^ This is mainly attributed to the critical role of amine hardeners, especially primary amines, in their production. Epoxy networks implementing primary amines rely on the theory that each primary amine ring opens two epoxy moieties and thus cross‐links the polymer chains. However, challenges emerge at this point. While the primary amine can always perform the initial attack, this may not hold true for the second ring‐opening reaction.^[^ ^4^, ^17^, ^58^, ^59^, ^60^ ^]^ The growing chains with increasing viscosity and steric hindrance around the formed secondary amine after the initial attack do not always allow the secondary amine to carry out a second attack. Consequently, the network structure cannot grow uniformly and symmetrically at every point. Therefore, the oligomeric structures formed after depolymerization do not consist of a well‐defined product but rather result in a mixture.^[^ ^17^, ^57^ ^]^ When a secondary amine is used as a hardener, the attack on the epoxy ring will be easier, since the amine is not attached to a growing chain. As a result, the network formed will grow more homogeneously. Therefore, the variety of products formed after depolymerization will be minimized. Considering all these facts, we envisioned an alternative pathway that integrates imine moieties with secondary amine functionalities, which can create a well‐grown epoxy network. Upon efficient acidic depolymerization, the well‐grown network would only produce well‐defined molecules due to the curing based on secondary amine functionality, allowing for the creation of original polymers by simply combining two distinct species as reactants. In our recent work, we have found that this unique design serves as a highly effective tool for multiple recycling cycles, maintaining the integrity and performance of the material throughout repeated processes.^[^ ^61^ ^]^

In addition to the chemical recycling of imine‐based epoxy networks, we were inspired by a recent study on the solubilization of imine‐based networks attributed to imine metathesis reactions in a very limited number of good solvents such as dichloromethane and chloroform.^[^ ^62^ ^]^ The selective solubilization and subsequent recasting of the polymeric network demonstrate the potential for solvent‐based recycling of imine‐based thermosets—a process currently limited to thermoplastic materials. This approach offers the advantage of preserving the integrity of all components of the composite material during recycling, while also being feasible under relatively mild conditions, thus presenting significant potential for the recycling of imine‐based epoxy composites.

Acidic depolymerization and solvent recycling also pave the way for carbon‐fiber recovery from carbon‐fiber‐reinforced imine‐based epoxy systems. Recovering carbon fiber from such composites is crucial due to the high cost and energy‐intensive nature of carbon fiber production.^[^ ^63^ ^]^ Recycling allows these high‐quality fibers to be repurposed for various applications.^[^ ^14^ ^]^

**Figure 1:**
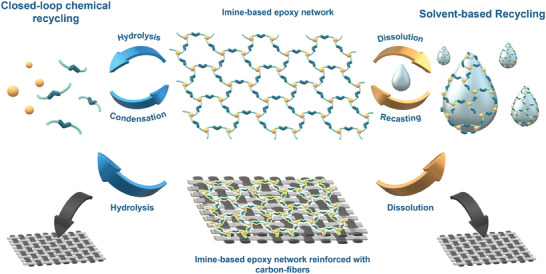
The representative closed‐loop chemical and solvent‐based recycling of epoxy/reinforced epoxy networks based on secondary amine imine hardener.

In this study, we aimed to develop a fully recyclable, high‐performance epoxy networks and composites based on DGEBA. Our approach integrates i) carbon fiber reinforcement, ii) excellent stability in neutral aqueous conditions, iii) high‐yield, acid‐catalyzed closed‐loop chemical recycling, and iv) efficient solvent‐based recycling of epoxy composites for the targeted separation of carbon fibers (**Figure** **1**). The key to meeting these criteria was the design and development of a liquid imine‐containing secondary amine hardener which offers a great potential for the circularity of epoxy thermosets and composites.

## 2 Results and Discussion

### 2.1 Monomer Synthesis and Characterization

**Figure 2:**
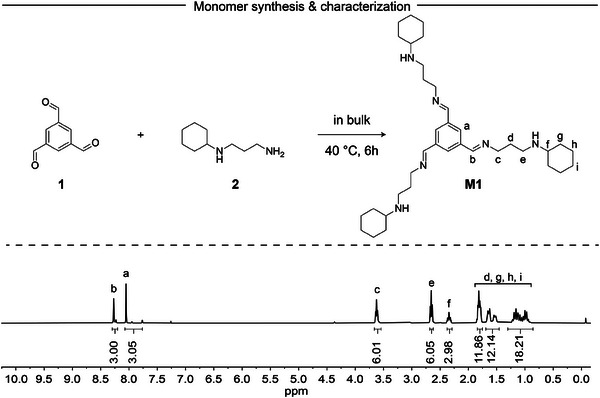
Synthesis scheme and ^1^H NMR spectrum of **M1**.

The synthesis of the imine‐containing secondary amine hardener **M1**, involved the reaction of benzene‐1,3,5‐tricarboxaldehyde (**1**) with *N*‐(3‐aminopropyl)cyclohexylamine (**2**) under bulk conditions at 40 °C for 6 h to yield a liquid monomer without the need for any purification process. During the condensation reaction, water molecules were produced and subsequently removed using sodium sulfate (Na_2_SO_4_). The structural characterization of **M1** was carried out using ^1^H and ^13^C NMR analyses, which were further supported by HSQC, HMBC, FTIR, and MALDI‐TOF. The ^1^H NMR spectrum (**Figure** **2**; Figure S1, Supporting Information) revealed a distinct signal at 8.27 ppm, attributed to the N═CH proton, confirming the formation of the imine bond. Additionally, the disappearance of aldehyde signals ≈10.24 ppm further corroborated the successful formation of the imine structure. The ^13^C NMR spectrum (Figure S2, Supporting Information) provided additional evidence, with a signal at 160.45 ppm corresponding to the iminyl carbon. To gain deeper insights into the molecular structure, HSQC, HMBC, and FTIR analyses were performed (Figures S3–S5, Supporting Information). The 2D NMR techniques provided detailed information on the position of atoms within the molecular structure. The results from HSQC and HMBC analyses confirmed the expected structure, supporting the formation of the desired imine molecule. Further structural confirmation was obtained through MALDI‐TOF analysis, which validated the synthesized monomer (Figure S6, Supporting Information). An important aspect of the synthesis was the prevention of intramolecular reactions that could lead to undesirable side products. The use of a bulky amine in the synthesis was strategic, as it hindered the attack on the iminyl carbon of the secondary amine present in the structure through intramolecular reactions.^[^ ^64^, ^65^ ^]^ This effectively prevented the formation of a potential hexahydropyrimidine moiety, ensuring the integrity of the desired imine hardener.^[^ ^64^ ^]^ Additionally, the compound demonstrated remarkable bench stability, remaining intact even when exposed to air for a duration of up to 4 months. On the other hand, utilizing less bulky amines, such as *N*‐methylethylenediamine and *N,N*‐dimethyldipropylenetriamine, as suspected, led to partial cyclization and the formation of imidazolidine (**A1**) and hexahydropyrimidine (**A2**) moieties respectively, due to intramolecular attacks, as supported by APT and 2D NMR analyses (Figures S7–S14, Supporting Information).

### 2.2 Curing Experiments for Epoxy Thermosets **P1** and **P1CF**

**Figure 3:**
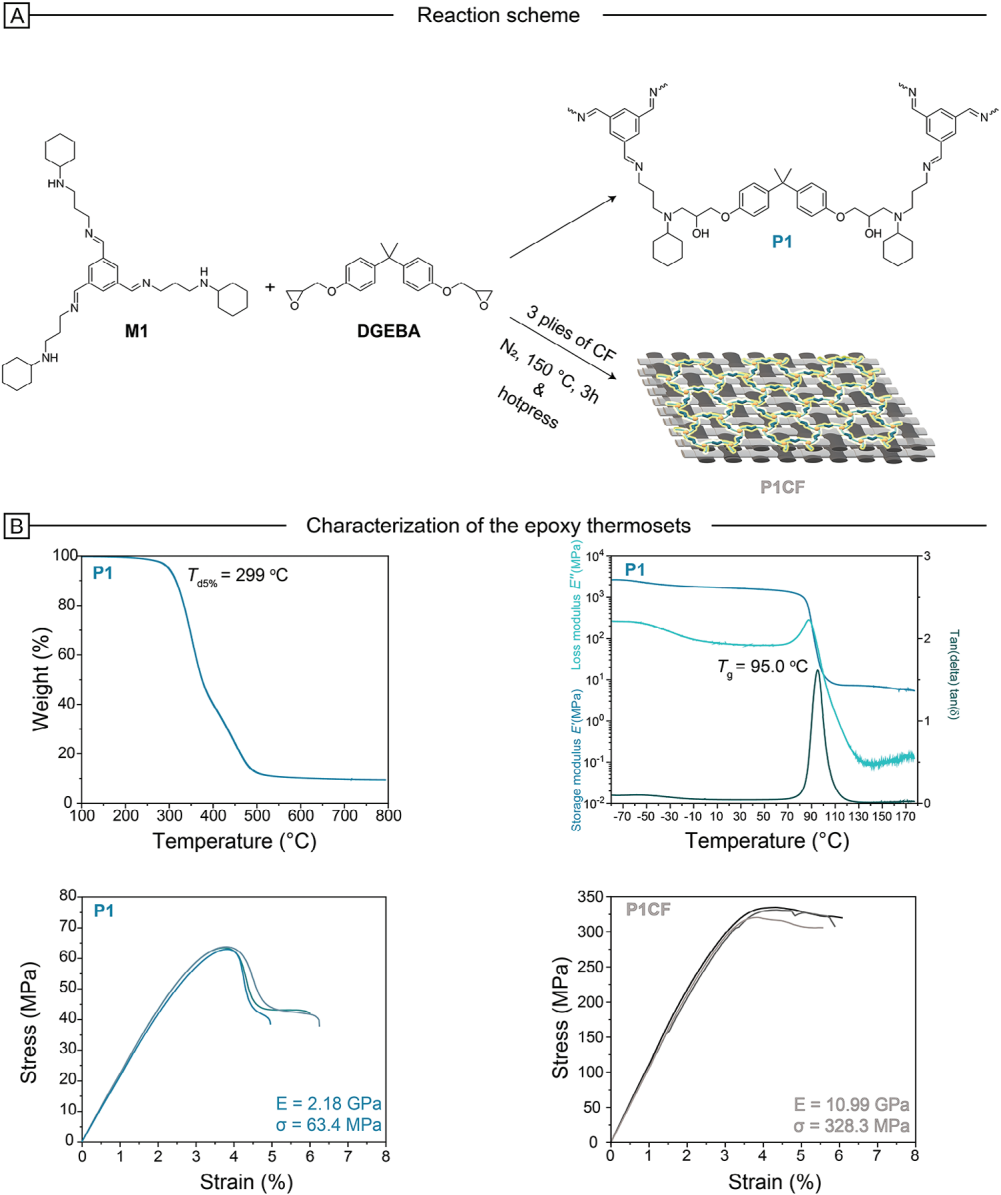
A) Reaction scheme of **P1** and **P1CF**; B) Thermal, thermomechanical, and mechanical properties of the polymers characterized by TGA, DMA, and tensile stress–strain tests.

To synthesize the epoxy network **P1**, we selected the widely‐used epoxy monomer DGEBA and reacted it with **M1**. Non‐isothermal differential scanning calorimetry (DSC) experiments were conducted at varying heating rates between 5 and 20 °C per minute to analyze the curing kinetics. Singular exothermic peaks were observed, and the peak values of these curves were employed in the Kissinger equation to calculate the activation energy for polymerization (Figure S15, Supporting Information). The activation energy was calculated as 54.7 kJ mol^−1^ for **P1**, aligning well with the activation energy values of classical epoxy systems.^[^ ^4^, ^66^, ^67^, ^68^ ^]^ The synthesis of the epoxy thermoset **P1** was carried out at 150 °C under a nitrogen atmosphere for 3 h. For the production of **P1CF**, the same reaction conditions were followed, with the addition of 3 plies of carbon fiber cloth for reinforcement. Subsequently, **P1CF** was pressed at 120 °C and 10 MPa pressure for 1 h (**Figure** **3**A).

### 2.3 The Characterization of **P1** and **P1CF** Epoxy Networks

The thermal and thermomechanical characteristics of **P1** were analyzed using thermogravimetric analysis (TGA), differential scanning calorimetry (DSC) (Figure S16, Supporting Information), dynamic mechanical analysis (DMA), and stress‐relaxation (**Table** **1**). Additionally, tensile stress–strain tests were conducted on both **P1** and **P1CF** to evaluate their mechanical performance (Figure 3B). The surface of **P1CF** was investigated using Scanning Electron Microscopy (SEM) analysis (Figure S17, Supporting Information). The thermal decomposition temperature at 5% weight loss (*T*_d5%_) for **P1** was observed at 299 °C. The glass transition temperature (*T*_g_) was measured at 71 and 95 °C, obtained from DSC and DMA, respectively. A stress relaxation experiment was conducted to examine the dynamic exchange behavior of the cross‐linked **P1** polymer. **P1** exhibited full stress relaxation, demonstrating the temperature‐dependent behavior typical for imine‐based networks. The activation energy of the bond exchange reaction was calculated to be 125.88 kJ mol^−1^ using the Arrhenius equation. (Figure S18, Supporting Information). The tensile test results demonstrated high performance for **P1**, with a Young's modulus of 2.18 GPa and a tensile strength of 63.4 MPa. These values are consistent with those reported for classical DGEBA‐based epoxy networks, aligning well with existing literature.^[^ ^4^, ^69^ ^]^ On the other hand, the reinforced network **P1CF** exhibited significantly higher performance as expected, with a Young's modulus of 10.99 GPa and tensile strength of 328.3 MPa. SEM analysis revealed that the carbon fibers were uniformly coated with the epoxy resin, suggesting a strong interaction between the two materials, likely facilitated by *π*–*π* interactions. The stability of **P1** was evaluated in various aprotic and protic solvents with different polarities, including n‐hexane, acetone, water, ethanol, methanol, ethyl acetate, acetonitrile, DMSO, and DMF (Figure S19, Supporting Information). Specimens were cut into identical rectangular dimensions and immersed in these solvents at room temperature for three days. Every 24 h, the samples were removed, the excess solvent was removed, and they were weighed. This procedure was repeated over a three‐day period, with weights recorded daily. Throughout the test, all specimens exhibited excellent stability in the selected solvents, including water which is an exceptional feature for imine‐based networks.^[^ ^70^ ^]^ The highest swelling ratio was observed in DMF at 123.1%, while n‐hexane and water showed no swelling behavior. Additionally, after drying the samples under vacuum post‐solvent treatment, the gel fractions were determined to be greater than 99% for all tested solvents, indicating the high stability of **P1**.

**Table 1:** Thermal and thermomechanical properties of **P1** and recycled polymers.

Polymer	*T*_d5%_ [°C]	*T*_d30%_[°C]	*T*_g_ [°C]	*E*_30_^′^[GPa]	Young's Modulus [GPa]	Tensile Strength [MPa]	Elongation at Break (%)
**P1**	299	347	71.4^a)^ /95.0^b)^	2.27	2.18 ± 0.05	63.4 ± 0.4	5.7 ± 0.7
**P1R**	299	342	99.2^b)^	2.50	2.48 ± 0.07	65.6 ± 1.9	3.7 ± 0.2
**P1S**	281	340	95.3^b)^	2.24	2.48 ± 0.08	63.8 ± 1.6	4.0 ± 0.2
**P1CFS**	284	345	101.3^b)^	2.26	2.58 ± 0.02	65.5 ± 1.5	4.3 ± 0.8

^a)^ Calculated from DSC analysis;

^b)^ Calculated from DMA analysis.

### 2.4 Closed‐Loop Chemical Recycling of **P1**

**Figure 4:**
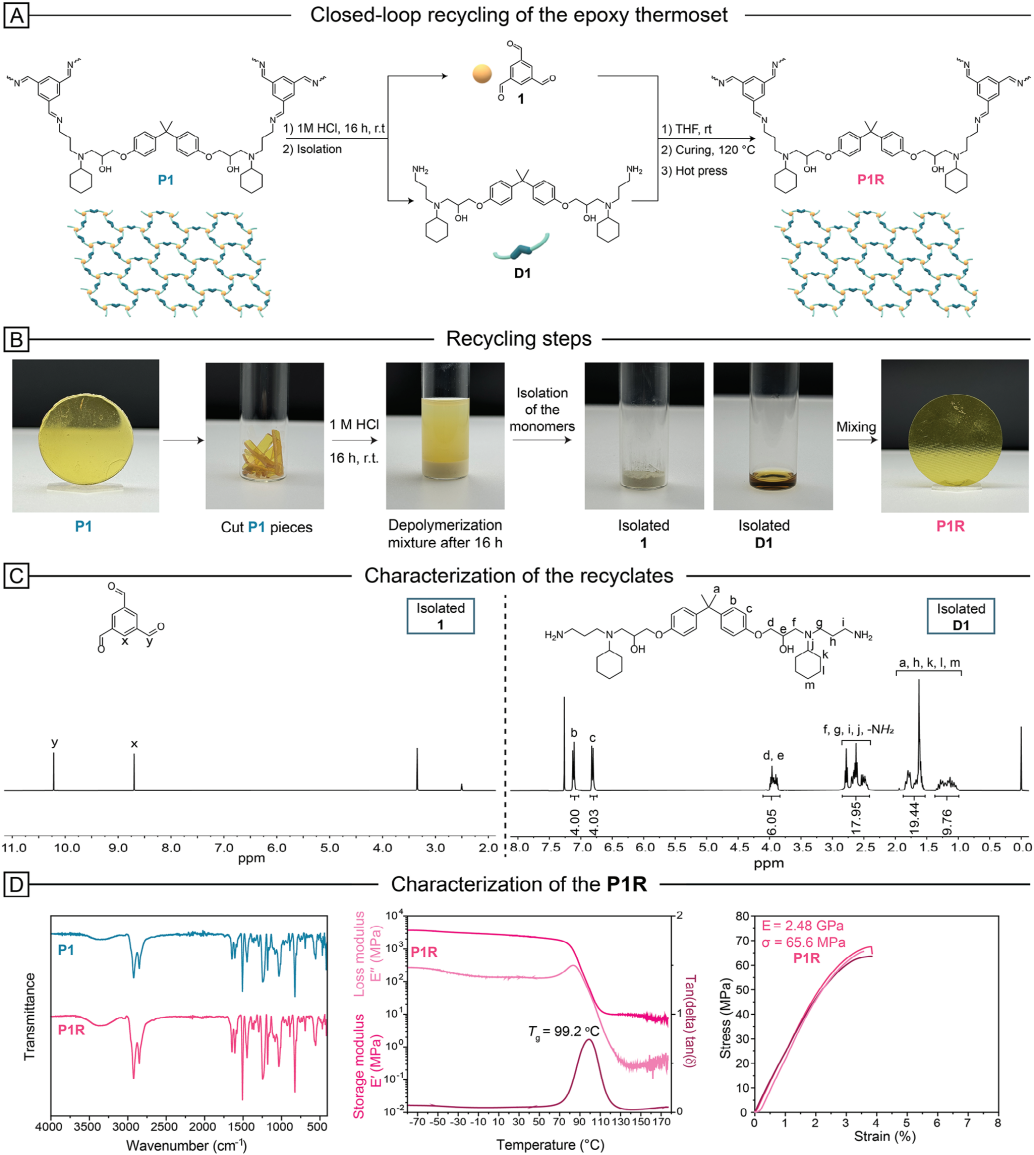
A) The chemical recycling process of **P1** to obtain the monomers **1** and **D1**, followed by the re‐synthesis of the recycled polymer **P1R**. B) Visual representation of the recycling procedure for **P1**. The ^1^H NMR spectra of the isolated monomers **1** and **D1**. Comparative analysis of the original polymer **P1** and the recycled polymer **P1R** including FTIR, DMA, and tensile stress–strain test.

For the acidic depolymerization of the epoxy network, 4.0 g of **P1** was weighed and immersed in 50 mL of 1 m HCl solution, then stirred at room temperature overnight (**Figure** **4**A,B). The precipitated product **1** was combined with the portion extracted from the solution using ethyl acetate, resulting in a 93% yield. The ^1^H NMR analysis of product **1** indicated ultimate purity, making it ready for use in recycling experiments (Figure 4C). The remaining aqueous phase was neutralized with 50 mL of 1 m NaOH and then extracted with DCM to obtain the DGEBA‐based depolymerization product **D1** with a 98% yield. The high purity of the monomer was confirmed by NMR, FTIR, and MS analyses (Figures S20–S22, Supporting Information). The aromatic signals of **D1** were detected at ≈7.13 and 6.83 ppm, while the CH_2_ signals of the cyclohexyl group appeared in the aliphatic region between 1.80 and 1.01 ppm. Depolymerization of **P1CF** under acidic conditions yielded the same depolymerization products as those obtained from **P1** (Figures S23 and S24, Supporting Information). The purity of these depolymerization products was confirmed by ^1^H NMR analysis. Additionally, SEM images demonstrated the integrity of the carbon fibers after acidic depolymerization (Figure 6B).

To fabricate **P1R** with the same performance as the original polymer **P1**, **D1** (3.0 g) and **1** (431.9 mg), with an amine/aldehyde ratio of 1.15:1.00, were dissolved in 15 mL of THF and transferred to a Teflon mold. The mixture was kept at room temperature for 4 h to allow the solvent to slowly evaporate and form a transparent film. The sample was then placed in a nitrogen oven set to 120 °C for 16 h. The remaining solvent was removed under reduced pressure with an additional treatment. To remove all bubbles formed during the evaporation of THF, **P1R** was hot‐pressed at 120 °C for 1 h at 10 MPa pressure (Figure 4A,B). The FTIR results of **P1R** displayed an identical pattern to those of **P1**. The appearance of CH═N stretching at 1645 cm^−1^ and the absence of CH═O stretching at 1680 cm^−1^ indicated the formation of the imines, confirming the completion of the reaction. The performance of the **P1R** was analyzed using TGA, DSC, DMA, measurements, and tensile stress–strain tests. The *T*_d5%_ value of the **P1R** was found to be 299 °C, which is identical to the virgin polymer (Figure S25, Supporting Information). The *T*_g_ value was calculated based on the maximum value of tanδ curve in the DMA as 99.2 °C (Table 1). The results of the tensile test indicated that the **P1R** polymer is high‐performing, with slightly higher values of Young`s modulus and tensile strength of 2.48 GPa and 65.6 MPa, respectively. These analyses confirmed that the polymer maintains its properties after the recycling process without any decrease in performance. Additionally, an attempt to recycle **P1R** using the same recycling procedure yielded **P1R2** with a Young's modulus of 2.35 GPa and a tensile strength of 66.3 MPa, further demonstrating the repeatability and consistency of the recycling procedure (Figure S26, Supporting Information).

### 2.5 Solvent‐Based Recycling of **P1** and **P1CF**

The solvent‐based recycling technique is typically viable only for thermoplastic materials due to their solubility behavior in different solvents.^[^ ^71^ ^]^ Thermosets, on the other hand, do not benefit from this feature because of their insoluble nature caused by highly cross‐linked architectures. However, recent discussions and studies have indicated the potential solubility of imine‐based networks in a limited number of good solvents such as CH_2_Cl_2_ and CHCl_3_.^[^ ^61^, ^72^, ^73^, ^74^ ^]^ The latest research has demonstrated that imine networks can exchange bonds with surrounding imine bonds in a swollen state due to the imine metathesis reaction in good solvents. This dynamic system ultimately leads to the formation of many small network clusters, thereby increasing their solubility.^[^ ^61^ ^]^ Based on this idea, we proposed a milder pathway to recover carbon fibers from epoxy composites and recycle the epoxy thermoset using our imine approach.

To evaluate this feature in our polymeric systems, we first immersed our **P1** polymer samples in CDCl_3_ to check the imine moiety. After 2 days, we obtained a clear solution without any insoluble particles. The ^1^H NMR results confirmed the intactness of the imine moieties, and no aldehyde formation was observed, indicating that no hydrolysis reaction occurred during this solubilization process (Figure S27, Supporting Information). In the next step, **P1** and **P1CF** samples were immersed in CH_2_Cl_2_ at room temperature and stirred for 3 days. Complete solubilization of the **P1** was achieved within this timeframe. However, due to the limited penetration of the solvent to the epoxy‐carbon fiber interface, an additional 5 days of stirring was required for the solubilization of the polymer matrix of **P1CF**, with the solvent being renewed daily.

**Figure 5:**
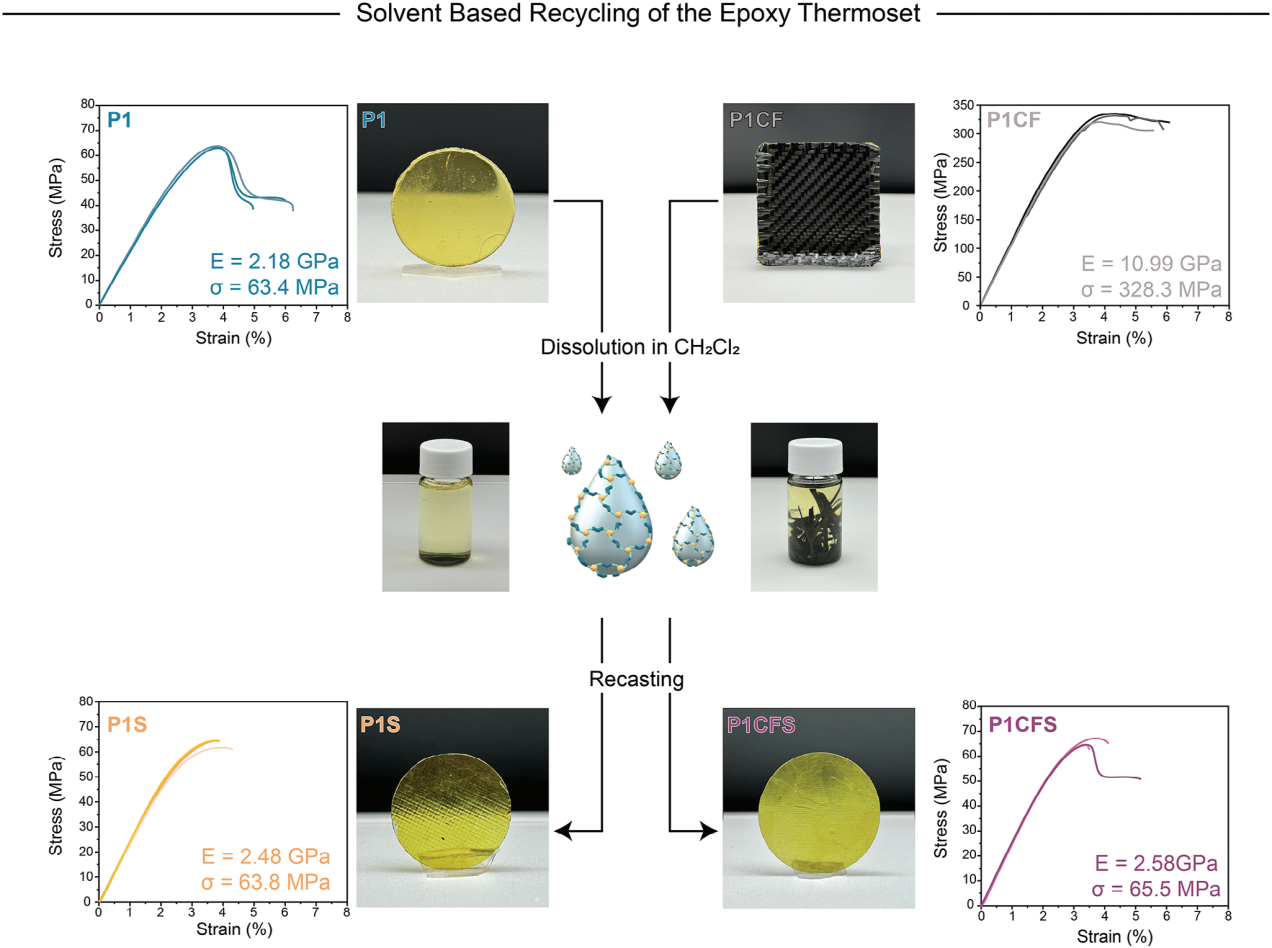
Illustrative representation of the solvent‐based recycling process of **P1** and **P1CF** into **P1S** and **P1CFS**, along with their respective tensile stress–strain test results.

**Figure 6:**
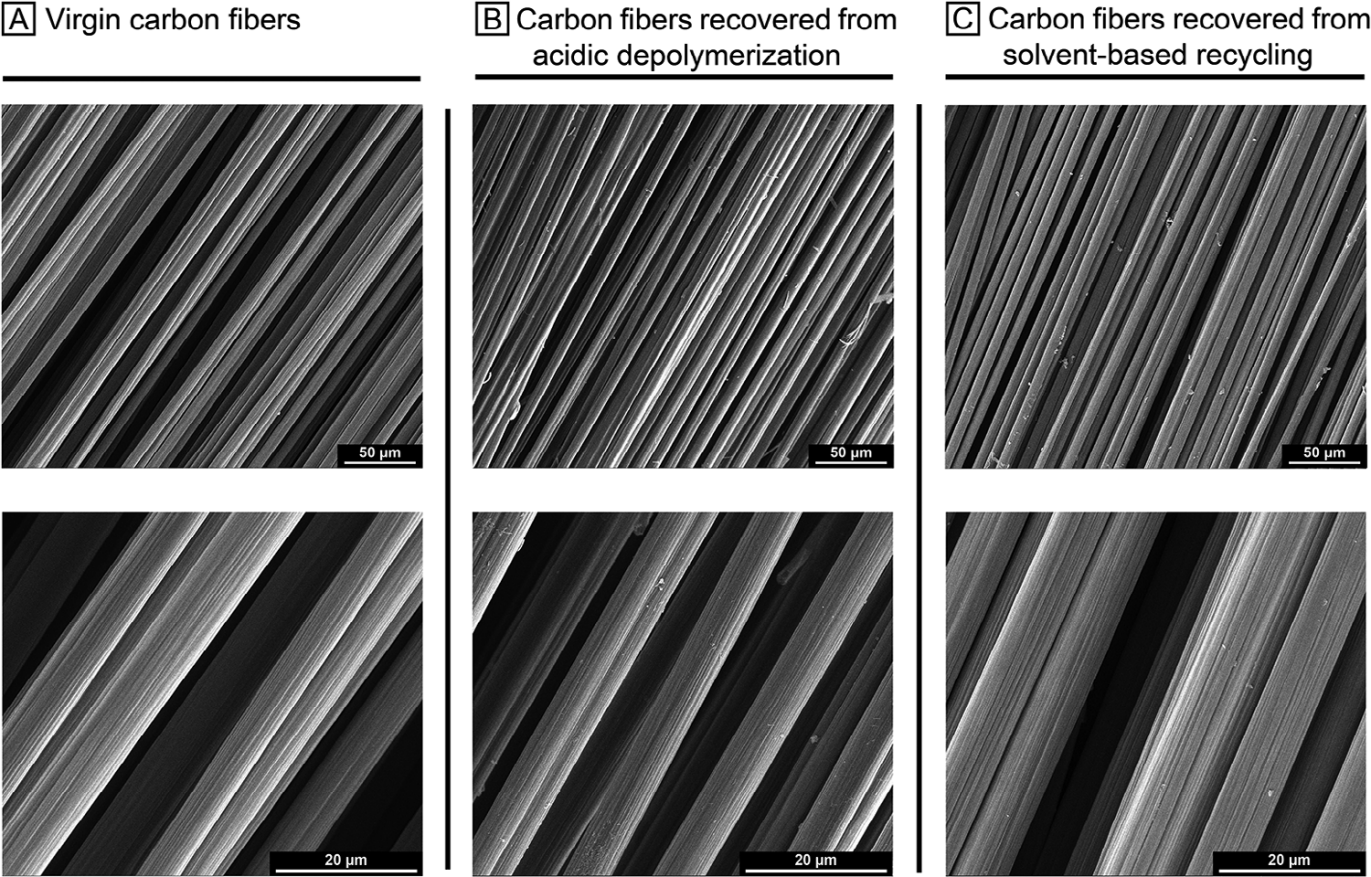
SEM images of A) virgin carbon fibers, B) carbon fibers recovered from acidic depolymerization, and C) carbon fibers recovered from solvent‐based recycling (top row: 50 µm scale, bottom row: 20 µm scale).

After this period, the solubilized epoxy network was transferred to separate Teflon molds and kept at room temperature until most of the solvent was evaporated, forming the polymeric films **P1S** and **P1CFS**. These polymeric mixtures were then placed in a nitrogen oven for 6 h at 120 °C. The remaining solvents were removed in a vacuum oven at 120 °C. Finally, **P1S** and **P1CFS** were hot‐pressed at 120 °C for 1 h at 10 MPa pressure to eliminate bubbles from the system. (**Figure** **5**) The FTIR results showed the intactness of the imine moieties, and hydrolysis was not observed as there was no aldehyde carbonyl stretching at 1680 cm^−1^ observed (Figures S28–S29, Supporting Information). Overall, **P1**, **P1S**, and **P1CFS** exhibited identical FTIR spectra, confirming the successful recycling of the **P1** and **P1CF** in solvent‐based conditions. TGA, DSC, DMA, and tensile stress–strain tests revealed remarkable similarities between the virgin polymer **P1** and the recycled polymers **P1S** and **P1CFS**. The *T*_d5%_ values of **P1S** and **P1CFS** were 281 and 286 °C (Figure S30, Supporting Information), respectively, while the *T*_d30%_ values were 340 and 345 °C, compared to 347 °C for **P1**. DMA results indicated that the *T*_g_ values of **P1S** and **P1CFS** were 95.3 and 101.3 °C, respectively (Figure S31, Supporting Information). Most importantly, the Young's modulus values were measured as 2.48 GPa for **P1S** and 2.58 GPa for **P1CFS**, with tensile strength values of 63.8 and 65.5 MPa, respectively. When all these results are evaluated together and compared with the initial polymer **P1**, it is evident that the solvent‐based recycling of both **P1** and **P1CF** was successfully achieved without compromising the performance of the unreinforced epoxy network. Additionally, SEM analysis of the recovered carbon fibers (**Figure** **6**C) demonstrated that the solvent‐based recycling method effectively preserved the integrity of the carbon fibers, highlighting the efficacy of our approach in maintaining the structural and functional properties of the reinforcing materials. This is further supported by Raman spectra (Figure S32, Supporting Information), which show nearly identical I_D_/I_G_ ratios for virgin, chemically recycled, and solvent‐based recycled fibers (2.28, 2.29, and 2.28, respectively) and confirm that the structural integrity of the carbon fibers remains intact after recycling.

## 3 Conclusion

In this study, we have successfully developed a fully recyclable, water‐stable, DGEBA‐based epoxy network with impressive thermal and mechanical properties, including a *T*_g_ of 95 °C, Young's modulus of 2.18 GPa, and tensile strength of 63.4 MPa. The carbon fiber‐reinforced network exhibited excellent performance, with a Young's modulus of 10.99 GPa and tensile strength of 328.3 MPa. Both materials demonstrated high performance and compatibility with closed‐loop chemical and solvent‐based recycling methods. Utilizing an imine‐containing secondary amine hardener, we created a robust thermoset with a well‐defined growth pattern. As an advantage, the developed monomer was liquid, which is crucial for industrial applications. Chemical recycling via acid‐catalyzed depolymerization yielded only well‐defined monomers with recovery ratios above 95%, confirmed by NMR, FTIR, and MS analyses. After recovering from depolymerization, the obtained monomers were reacted to form **P1R**, which exhibited properties nearly identical to the original polymer **P1**, with a *T*_g_ of 99.2 °C, Young's modulus of 2.48 GPa, and tensile strength of 65.6 MPa. Solvent‐based recycling was achieved by complete solubilization of the epoxy networks **P1** and **P1CF** in CH_2_Cl_2_, followed by recovery and reformation into polymer films **P1S** and **P1CFS**. TGA, DSC, DMA, and tensile stress–strain tests demonstrated that the recycled polymers retained thermal and mechanical properties comparable to the virgin polymer, with *T*_g_ values of 95.3 and 101.3 °C, Young's modulus of 2.48 and 2.58 GPa, and tensile strength of 63.8 and 65.6 MPa, respectively (Table 1). Selective solubilization allowed for the mild recovery of carbon fibers, with SEM analysis and Raman spectroscopy confirming their integrity.

These findings highlight the potential of imine‐containing secondary amine hardeners in different recycling methods to effectively integrate DGEBA monomers and carbon fibers into the circular polymer stream without losing performance. This approach is promising for industrial applications, including wind turbine blades, coatings, and adhesives, by providing a sustainable and high‐performance solution.

## Conflict of Interest

The authors declare no conflict of interest.

## Supporting information



Supporting Information

## Data Availability

The data that support the findings of this study are available from the corresponding author upon reasonable request.
