# Supplementary material for: Chemical and Solvent‐Based Recycling of DGEBA‐Based Epoxy Thermoset and Carbon‐Fiber Reinforced Epoxy Composite Utilizing Imine‐Containing Secondary Amine Hardener
Source: Macromol Rapid Commun. 2024 Nov 9;46(2):2400678. doi: 10.1002/marc.202400678 (PMC11756867; doi:10.1002/marc.202400678)
Supplement: Supplementary file 1 — Supporting Information [file MARC-46-2400678-s001.pdf]

Macromolecular Rapid Communications

Supporting Information

for *Macromol. Rapid Commun.*, DOI 10.1002/marc.202400678

Chemical and Solvent-Based Recycling of DGEBA-Based Epoxy Thermoset and Carbon-Fiber Reinforced Epoxy Composite Utilizing Imine-Containing Secondary Amine Hardener

*Özgün Dağlar, Tankut Türel, Christos Pantazidis and Željko Tomović**

Supporting Information

Chemical and Solvent-Based Recycling of DGEBA-Based Epoxy Thermoset and Carbon-Fiber Reinforced Epoxy Composite Utilizing Imine-Containing Secondary Amine Hardener

Özgün Dağlar, Tankut Türel, Christos Pantazidis, and Željko Tomović*

Dr. Özgün Dağlar, Dr. Tankut Türel, Dr. Christos Pantazidis, Prof. Dr. Ž. Tomović
Polymer Performance Materials Group, Department of Chemical Engineering and Chemistry, and Institute for Complex Molecular Systems (ICMS), Eindhoven University of Technology, 5600 MB Eindhoven, The Netherlands.
E-mail: z.tomovic@tue.nl

Table of Contents

Experimental section ... 3
1. Materials ... 3
2. Methods ... 3
3. Design and Synthesis of the Monomer ... 5
3.1 Synthesis of Imine-Containing Secondary Amine Hardener (**M1**) ... 5
3.2 Reaction of **1** with *N*-methylethylenediamine (**A1**) ... 8
3.3 Reaction of **1** with *N*,*N*-dimethyldipropylenetriamine (**A2**) ... 11
4. Curing Kinetic Studies of P1 ... 14
5. The Curing of the Polymers ... 14
5.1 The Curing of **P1** ... 14
5.2 The Curing of **P1CF** ... 15
6. Stress Relaxation Test and Activation Energy Analyses of the Bond Exchange Reaction ... 16
7. Stability Test, Swelling Ratio, and Gel Fraction Calculation ... 16
8. Depolymerization of P1 ... 18
9. Closed-Loop Chemical Recycling ... 21
10. Solvent-Based Recycling of P1 (P1S) ... 22
11. Solvent-Based Recycling of P1CF (P1CFS) ... 23

Experimental section

1. Materials

N-(3-aminopropyl)cyclohexylamine was purchased from TCI Europe B.V. Bisphenol A diglycidyl ether (DGEBA, D.E.R 332, Mw:340.4g/mol), *N*,*N*-dimethyldipropylenetriamine, hydrochloric acid (1.0 N), sodium hydroxide (1.0 N), sodium sulfate were purchased from Merck life science NV. Benzene-1,3,5-tricarboxaldehyde, *N*-methylenediamine, and were purchased from BLDpharm. Chloroform-*d* (D, 99.5%) and dimethyl sulfoxide-*d6* (D, 99.9%) were purchased from Cambridge Isotope Laboratories, Inc. Ethanol (AR), methanol (AR), acetone (AR), ethyl acetate (AR), n-hexane (AR), acetonitrile (HPLC), dimethyl sulfoxide (HPLC, DMSO), dimethylformamide (AR, DMF), diethylether (AR), dichloromethane (AR, CH_2Cl_2), chloroform (AR, $CHCl_3$) were purchased from Biosolve B.V. Carbon fibers (S-CF-22-240-125) were purchased from EasyComposites.

2. Methods

The ^{1}H NMR, ^{13}C NMR, APT, HSQC, and HMBC spectra were recorded on a Bruker UltraShield (400 MHz) using $CDCl_3$ and DMSO-d6 as solvents.

Mass spectroscopy of the compounds was performed with a Bruker Autoflex III TOF/TOF MALDI analyzer and LCQ Fleet ESI-MS (Thermo Fisher Scientific).

The FTIR spectra were recorded on a Thermo Scientific NICOLET iS20 FTIR spectrometer as an average of 8 scans over the wavenumber range of 450−4000 cm^{-1}.

Raman spectra were recorded using a Confocal Raman microscope WITec WMT 50, equipped with a 532 nm laser set at 10% laser power. The I_D/I_G ratios were calculated by integrating the area under the respective peaks.

TGA analyses were carried out using a TGA550 instrument (TA Instruments), where samples weighing 5 to 10 mg were heated from 100 to 800 °C under a nitrogen (N_2) atmosphere.

DSC measurements were performed on a TA Instruments Q2000. The samples(5−15 mg) were placed in an Aluminum-Hermetic pan. The experiments were carried out from −80 to 200 °C at a rate of 10 °C/min under N_2 atmosphere. Glass transition temperatures (T_g) were determined by taking the midpoint of the reversible endotherm of the second heating. The curing kinetics

of the polymers were measured using non-isothermal differential scanning calorimetry at heating rates of 5, 10, 15, and 20 °C/min under a nitrogen (N_2) atmosphere.

Dynamic mechanical analysis (DMA) measurements were performed on a TA Instruments DMA850. The experiments were carried out on samples with dimensions of 16 mm in length, 4 mm in width, and a measured thickness of around 0.8 mm. The temperature range for the experiments was from −80 to 180 °C, with a heating rate of 3 °C/min under an oscillatory strain of 0.1% and a frequency of 1 Hz. The glass transition temperature (T_g) was recorded as the maximum value of tan δ.

Stress relaxation analysis was conducted using a Discovery HR20 (TA Instrument). The relaxation modulus (G(t)) was monitored over various time intervals with a constant applied strain of 1% at temperatures ranging between 130 and 160 °C. The relaxation modulus (G) was normalized against its initial value ($G_{0.1}$). The activation energy (E_a) for the bond exchange reaction was subsequently determined using the Arrhenius equation:

$$\tau^*(T) = \tau_0 \ \exp\left(\frac{E_a}{RT}\right)$$

where τ* represents the relaxation time, defined as the point at which the modulus relaxes to 1/e, τ_0 is the characteristic relaxation time at infinite temperature, E_a is the experimentally determined activation energy (kJ/mol), R is the universal gas constant (8.314 J $K^{-1}mol^{-1}$), and T is the absolute temperature (K).

Tensile tests were conducted using a Zwick/Roell Intelligent testing machine equipped with a 2.5 kN load cell. For the non-reinforced dumbbell-shaped specimens, the effective length was 12 mm, the width was 2 mm, and the measured thickness was approximately 0.9 mm. For the carbon fiber-reinforced materials, the dimensions were similarly an effective length of 12 mm, a width of 2 mm, and a measured thickness of around 1.3 mm. All tests were carried out at a constant strain rate of 50 mm/min.

Scanning electron microscopy (SEM, Phenom ProX) was used to investigate the surface of the carbon fibers at an acceleration voltage of 10 kV.

The viscosity of **M1** was measured using a Physica MCR 301 viscometer (Anton Paar GmbH) equipped with a 50 mm plate-plate setup and a gap of 0.4 mm. A 1 mL sample of **M1** was applied to the system, and viscosity was determined through a shear rate sweep at 1 s^{-1} at 23 °C.

3. Design and Synthesis of the Monomer

Monomer synthesis was successfully achieved through the reaction between benzene-1,3,5-tricarboxaldehyde (**1**) and *N*-(3-aminopropyl)cyclohexylamine (**2**), yielding the liquid three-functional imine-based epoxy hardener **M1**. Comprehensive NMR and MS analyses confirmed the target molecule (**Figures S1-S6**). In contrast, employing *N*-methylethylenediamine and *N*,*N*-dimethyldipropylenetriamine resulted in the formation of imidazolidine and hexahydropyrimidine-based molecules **A1** and **A2**, respectively, due to the intramolecular reaction of secondary amines with iminyl carbons. Structural confirmations were made by NMR analysis, particularly using APT, HSQC, and HMBC analyses. In the APT spectrum, the positive C signals at 84.62 ppm for **A1** (**Figure S13**) and 81.68 ppm for **A2** (**Figure S22**) indicate the bridge protons belonging to the imidazolidine and hexahydropyrimidine structures, respectively. Additionally, the characterization of carbon atoms adjacent to the target carbons on HMBC analyses confirmed the intramolecular cyclization.

3.1 Synthesis of Imine-Containing Secondary Amine Hardener (**M1**)

N-(3-aminopropyl)cyclohexylamine (**2**) (15.0 g, 96 mmol) was weighed into a 250 mL round bottom flask. Benzene-1,3,5-tricarboxaldehyde (**1**) (5.2 g, 32 mmol) was added directly to the flask at room temperature. The mixture was stirred at 40 °C until homogenized. After achieving homogeneity, the resulting yellow mixture was left to stir at room temperature for 6 hours. Upon completion of the stirring period, 80 mL of CH_2Cl_2 was added to the viscous mixture to obtain a two-phase mixture. Na_2SO_4 was then added to the mixture and stirred for an additional 30 minutes. The drying agent was filtered off, and the remaining mixture was concentrated under reduced pressure at 40°C to yield a yellow viscous monomer with quantitative yield. ^{1}H NMR (400 MHz, $CDCl_3$, δ) 8.27-8.23 (m, 3H, N=C*H*), 8.05-7.77 (m, 3H, Ar*H*), 3.62 (m, 6H, NCH_2), 2.66 (m, 6H, NHCH_2), 2.33 (m, 3H, NHC*H*), 1.82-0.92 (m, 36H, CH_2 protons of cyclohexyl and $NCH_2CH_2CH_2$). ^{13}C NMR ($CDCl_3$, δ) 160.45, 159.94, 137.10, 129.30, 59.95, 56.85, 45.06, 33.66, 31.50, 26.20, 25.08. MS (MALDI-TOF) *m*/*z*: $[M + H]^+$ calculated for $C_{36}H_{61}N_6^+$ 577.93, found 577.48. The viscosity of the resulting monomer was measured to be 7265 mPa·s at a shear rate of 1 s^{-1}.

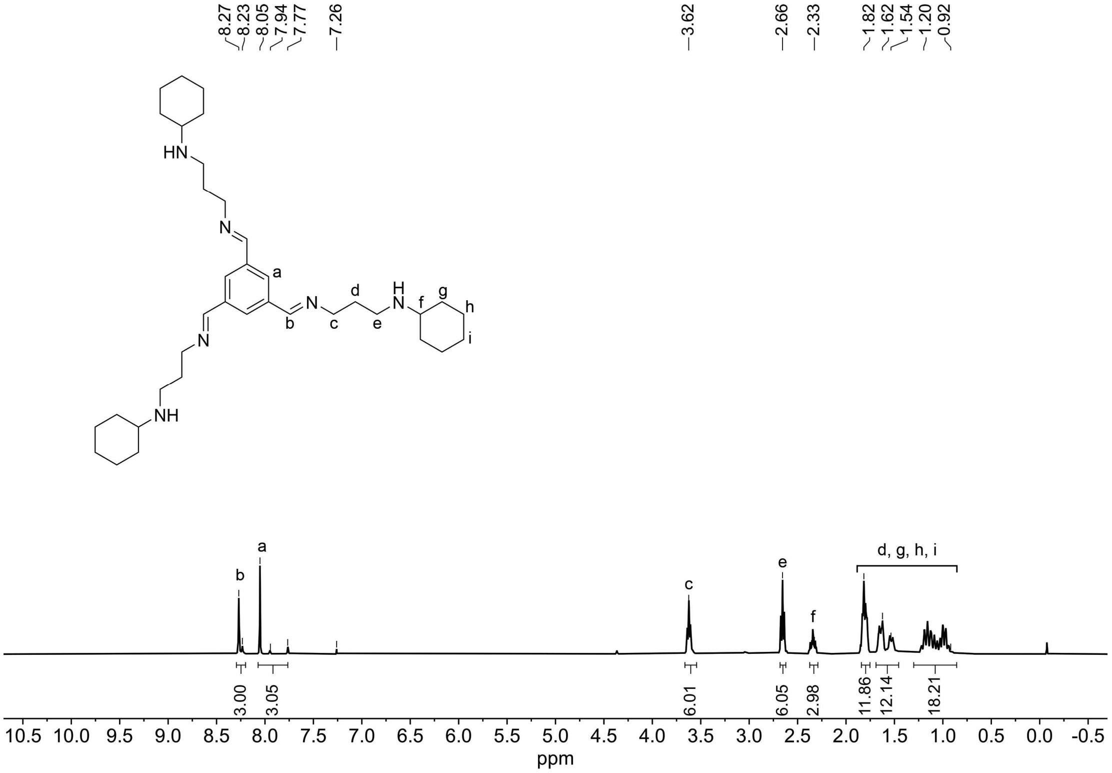

Figure S1. ^{1}H NMR spectrum of **M1** ($CDCl_3$, 400 MHz).

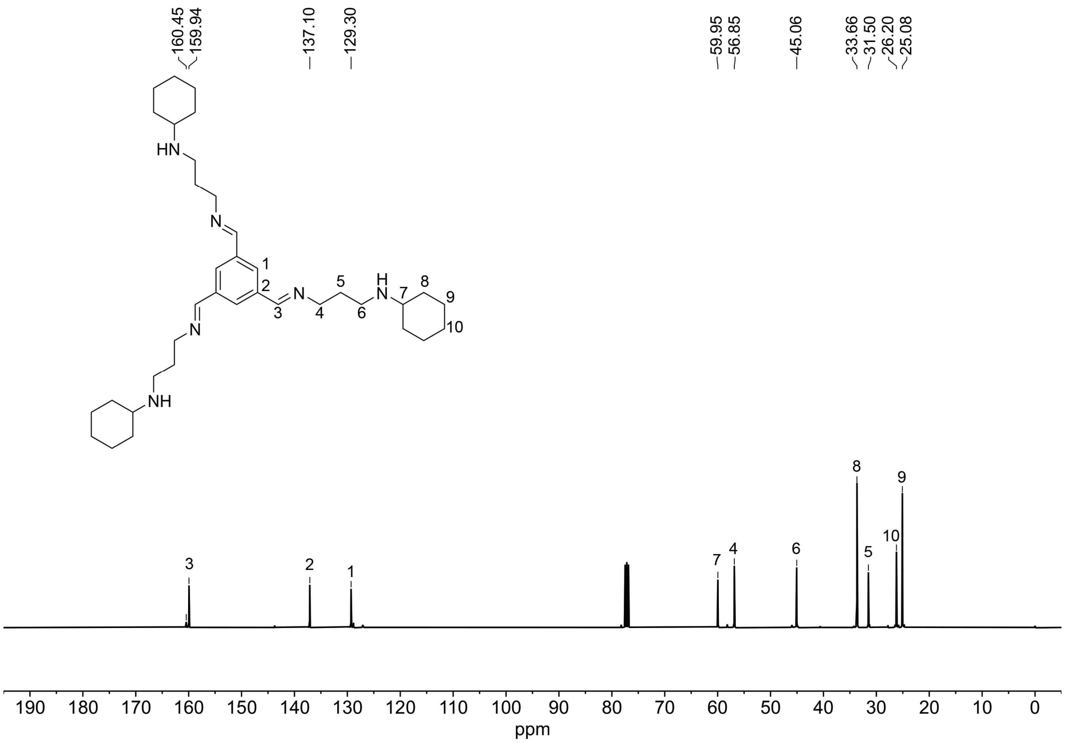

Figure S2. ^{13}C NMR spectrum of **M1** ($CDCl_3$, 101 MHz).

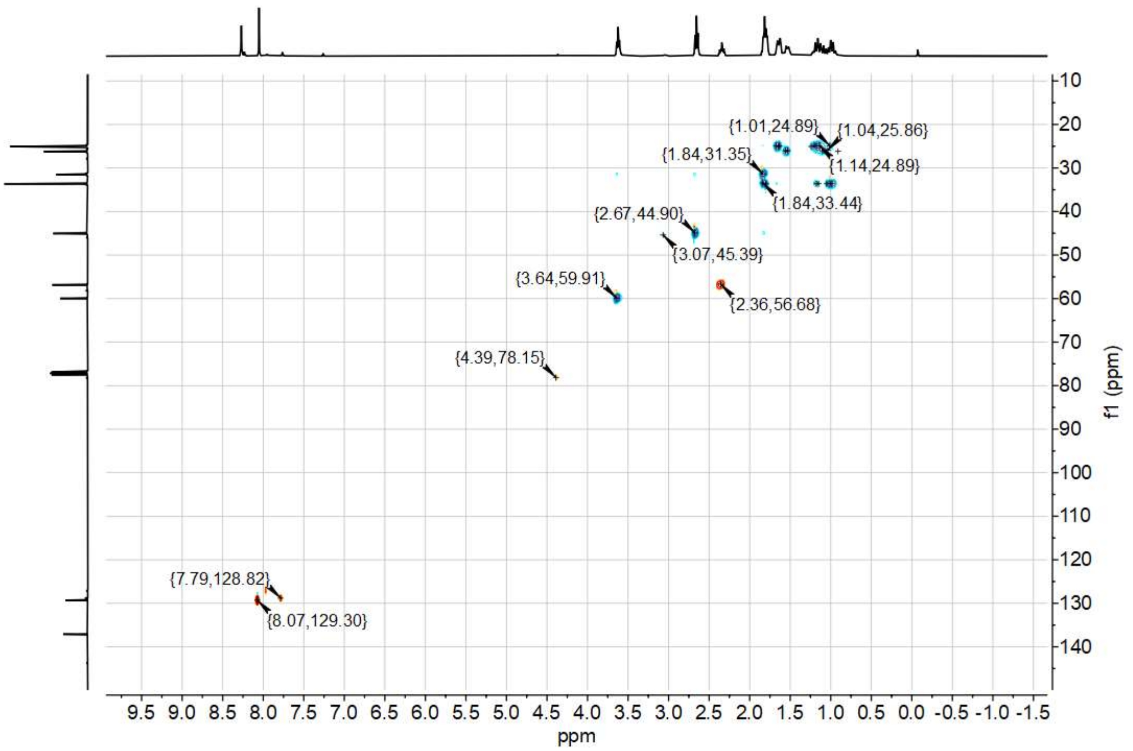

Figure S3. HSQC spectrum of **M1** in $CDCl_3$.

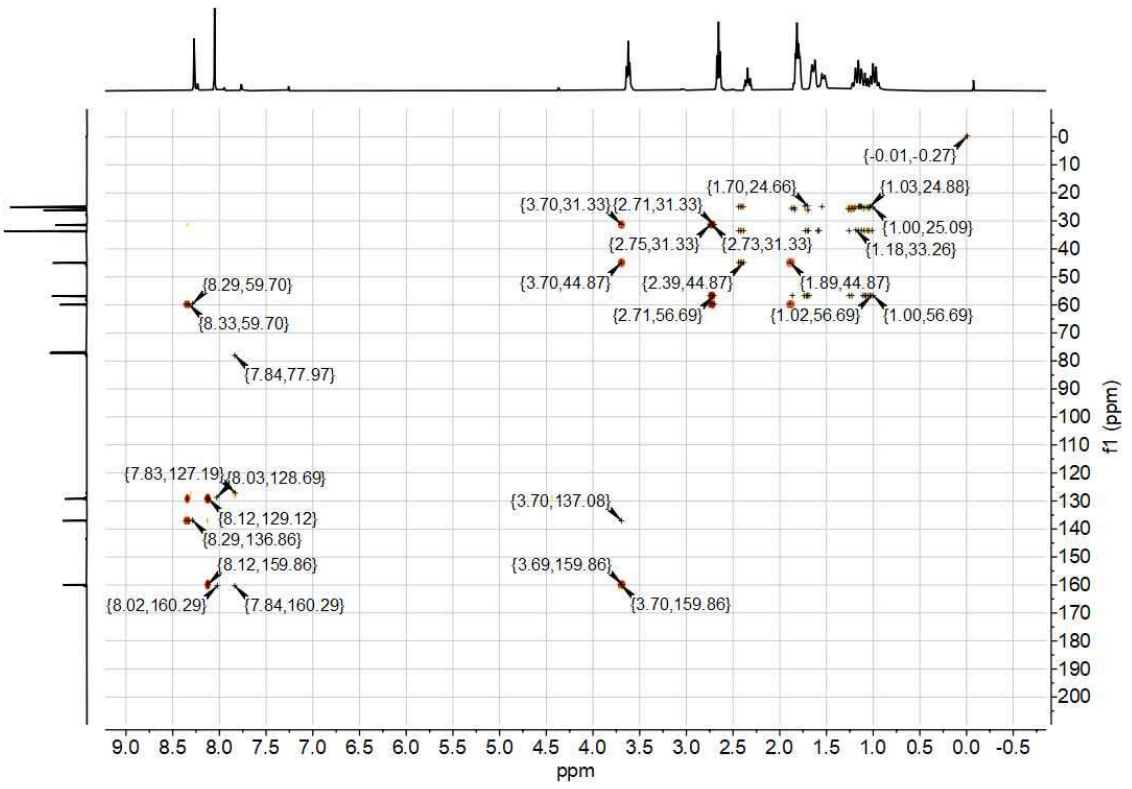

Figure S4. HMBC spectrum of **M1** in $CDCl_3$.

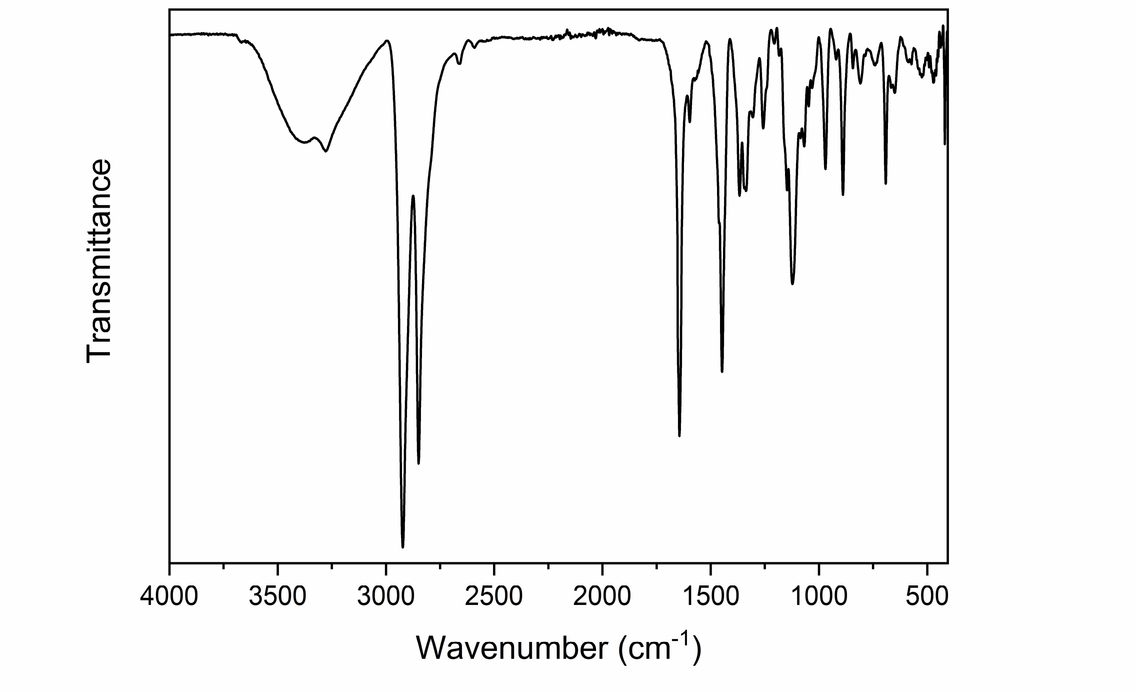

Figure S5. FTIR spectrum of **M1**.

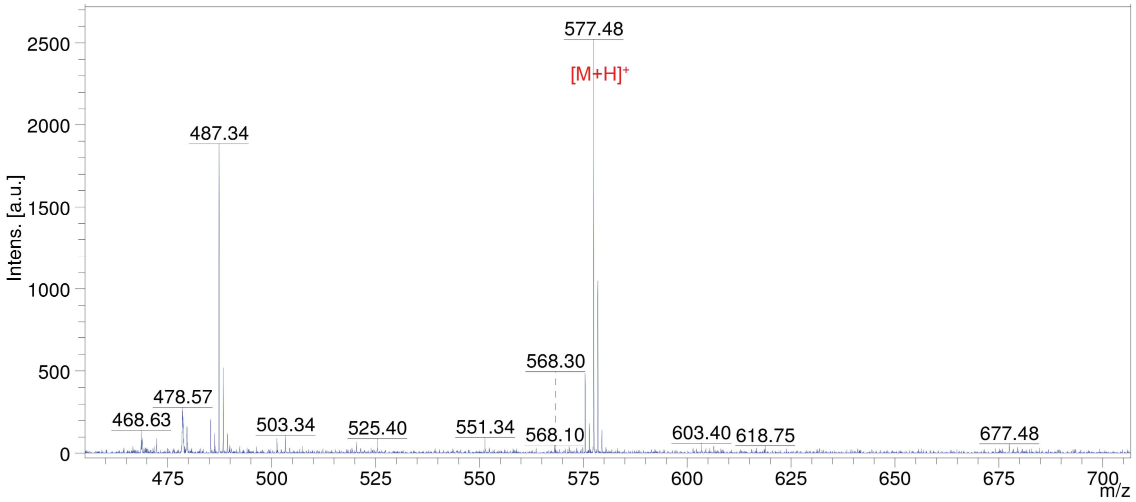

Figure S6. MALDI-TOF spectrum of **M1**.

3.2 Reaction of **1** with *N*-methylethylenediamine (**A1**)

0.74 g (10 mmol) of *N*-methylethylenediamine was weighed in a 100 mL round bottom flask and dissolved in 20 mL of THF. The flask was then placed in an ice bath. Over the course of 1 hour, 20 mL of THF containing 0.54 g (3.3 mmol) of **1** was added dropwise to the solution in the ice bath. The resulting mixture was allowed to stir at room temperature for 18 hours. The

following day, THF was removed under vacuum at 40 °C. The resulting compound was dissolved in CH_2Cl_2 and dried over Na_2SO_4. The drying agent was filtered, and the remaining solution was evaporated to yield the final product (yield = 0.86 g, 75.0 %). ^{1}H NMR ($CDCl_3$, *δ*) 8.20 (br, 1H, N=C*H*), 7,89-7.39 (m, 3H, Ar*H*), 3.75-3.60 (m, 4H, HC=NC*H*$_2$ and NC*H*NH), 3.18-2.29 (m, 13H, NC*H*$_2$, NHC*H*$_2$ and NHC*H*$_3$), 2.15-2.07 (m, 6H, NC*H*3). ^{13}C NMR ($CDCl_3$, *δ*) 161.81, 161.40, 141.21, 140.82, 136.77, 129.23, 127.49, 126.88, 84.60, 84.18, 61.56, 60.87, 59.36, 55.37, 51.80, 44.42, 38.57, 36.13.

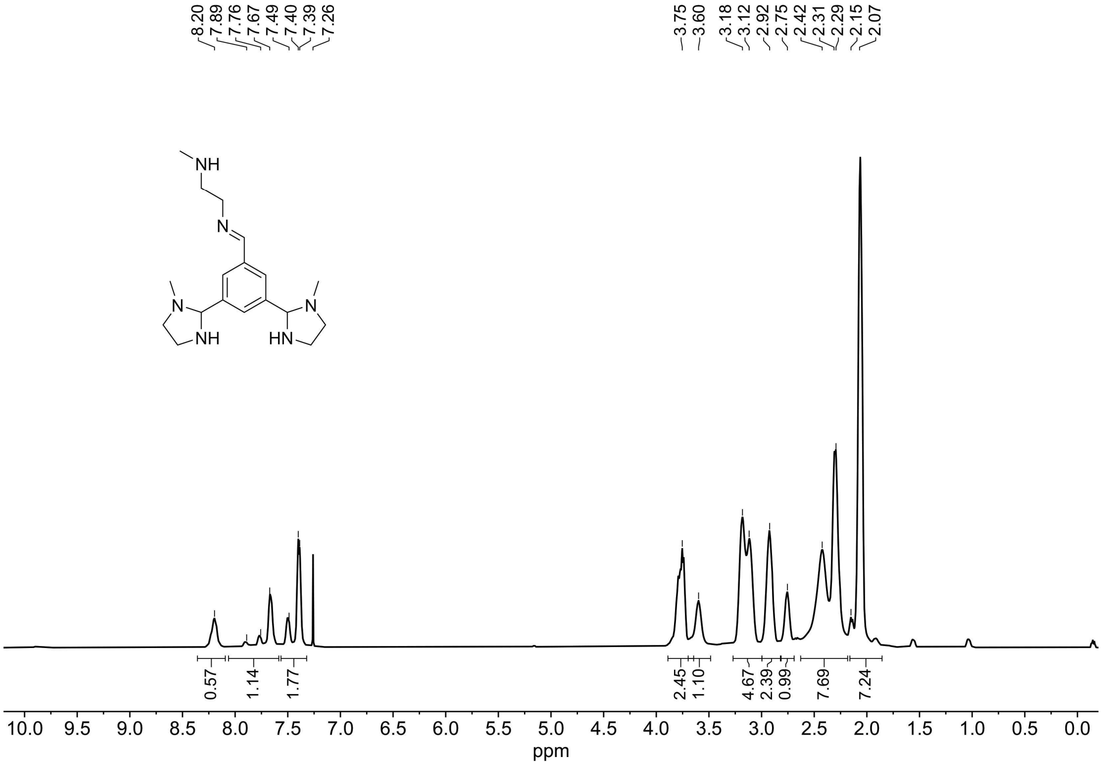

Figure S7. ^{1}H NMR spectrum of **A1** ($CDCl_3$, 400 MHz).

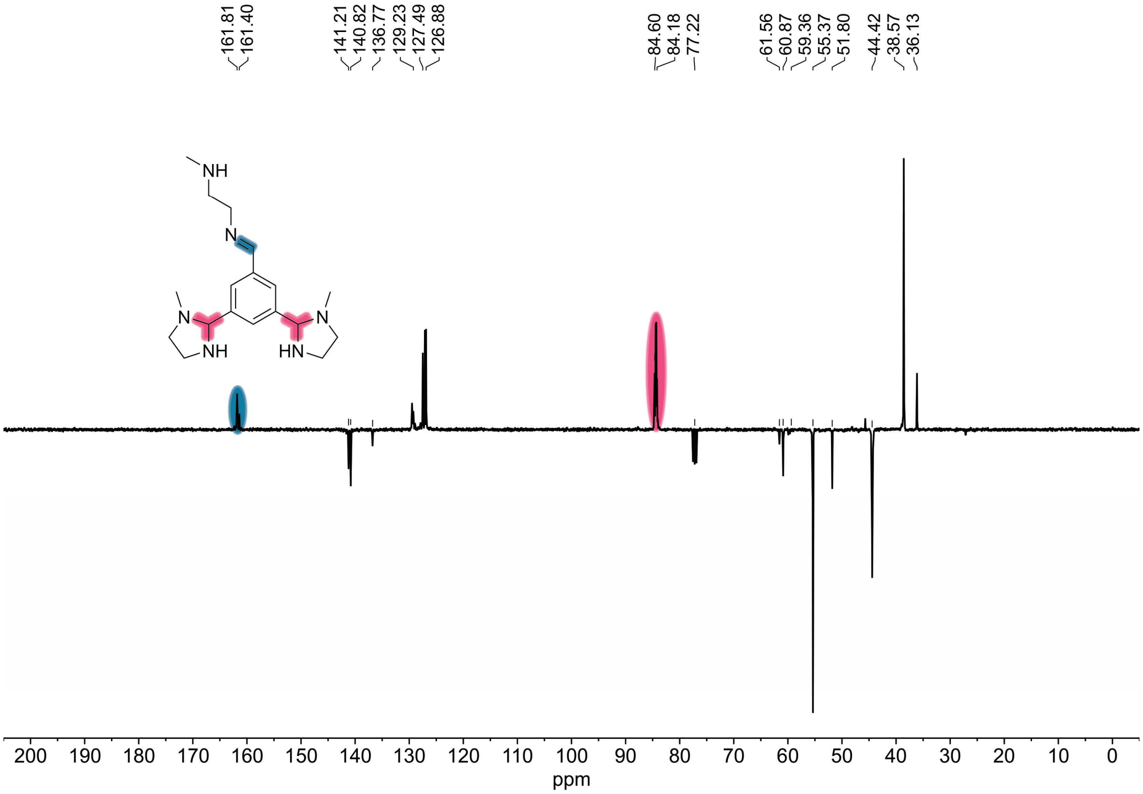

Figure S8. APT spectrum of **A1** in $CDCl_3$.

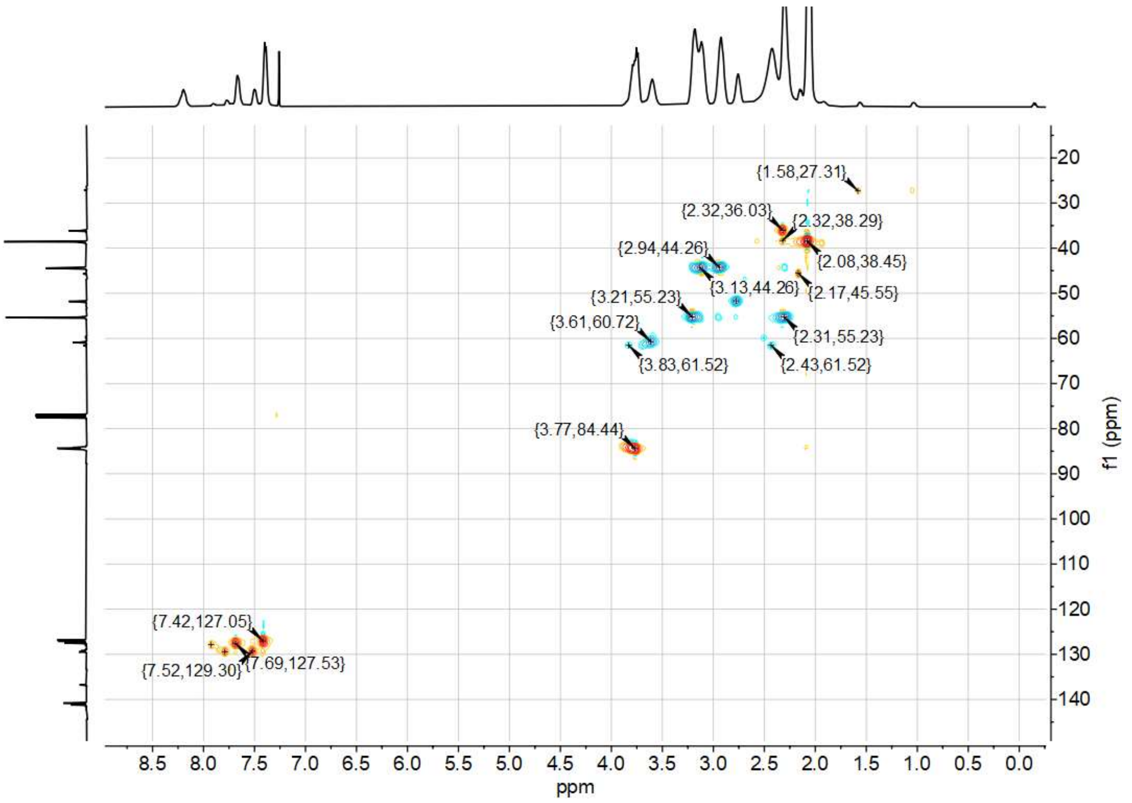

Figure S9. HSQC spectrum of **A1** in $CDCl_3$.

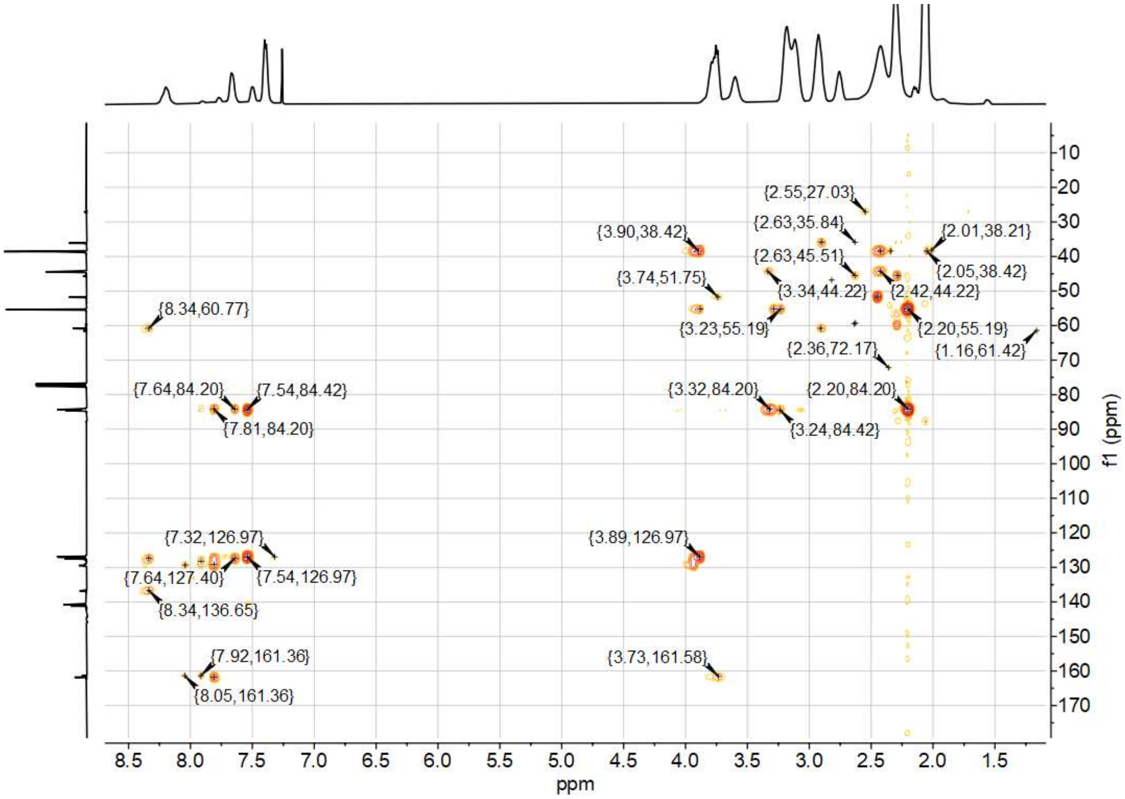

Figure S10. HMBC spectrum of **A1** in $CDCl_3$.

3.3 Reaction of **1** with *N*,*N*-dimethyldipropylenetriamine (**A2**)

1.59 g (10 mmol) of *N,N*-dimethyldipropylenetriamine was weighed in a 100 mL round bottom flask and dissolved in 20 mL of THF. The flask was then placed in an ice bath. Over the course of 1 hour, 20 mL of THF containing 0.54 g (3.3 mmol) of **1** was added dropwise to the solution in the ice bath. The resulting mixture was allowed to stir at room temperature for 18 hours. The following day, THF was removed under vacuum at 40 °C. The resulting compound was dissolved in CH_2Cl_2 and dried over Na_2SO_4. The drying agent was filtered, and the remaining solution was evaporated to yield the final product (yield = 1.68 g, 86.1 %). ^{1}H NMR ($CDCl_3$, δ) 8.02-7.97 (m, 1H, N=C*H*), 7,70-7.13 (m, 3H, Ar*H*), 3.71-3.35 (m, 4H, HC=NCH_2 and NC*H*NH), 2.99-2.02 (m, 22H, NCH_2 and NHCH_2), 1.91-1.81 (m, 9H, NCH_3), 1.67-1.21 (m, 10H, $CH_2CH_2CH_2$). ^{13}C NMR ($CDCl_3$, δ) 161.15, 159.89, 143.12, 142.59, 136.69, 128.62, 126.67, 126.10, 82.05, 81.10, 59.99, 58.06, 57.54, 51.93, 48.10, 47.35, 46.09, 45.09, 30.49, 27.80, 27.19, 26.53, 24.50, 24.06.

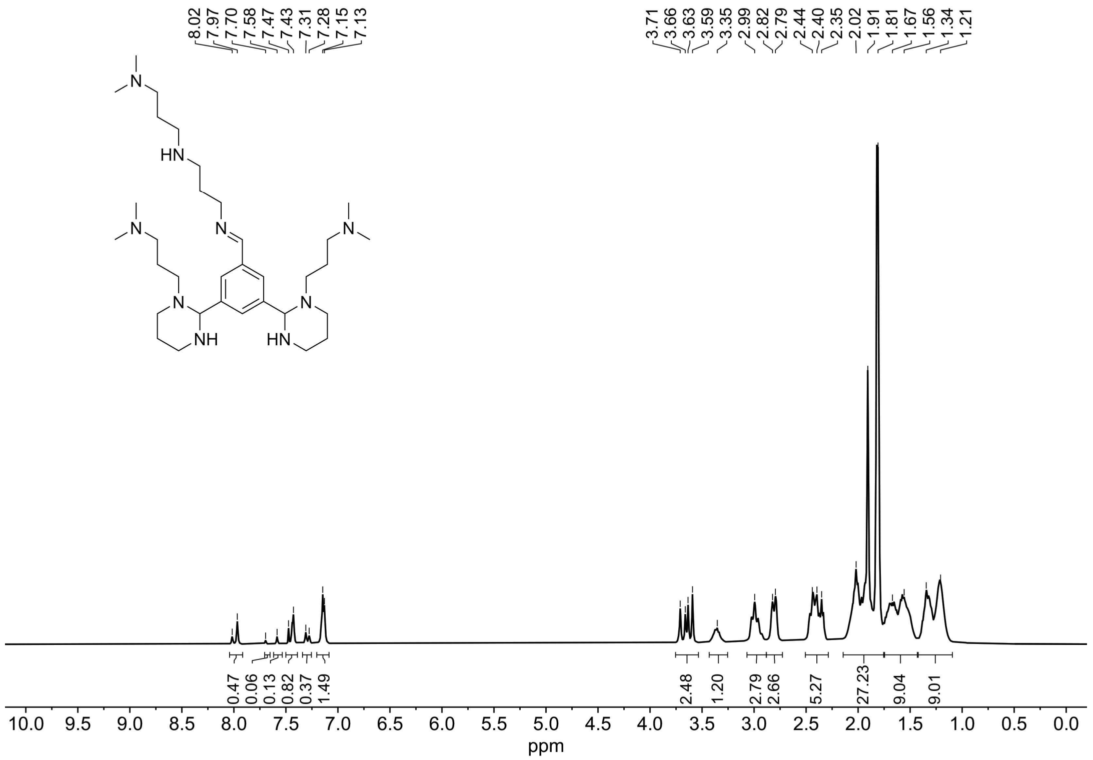

Figure S11. ^{1}H NMR spectrum of **A2** ($CDCl_3$, 400 MHz).

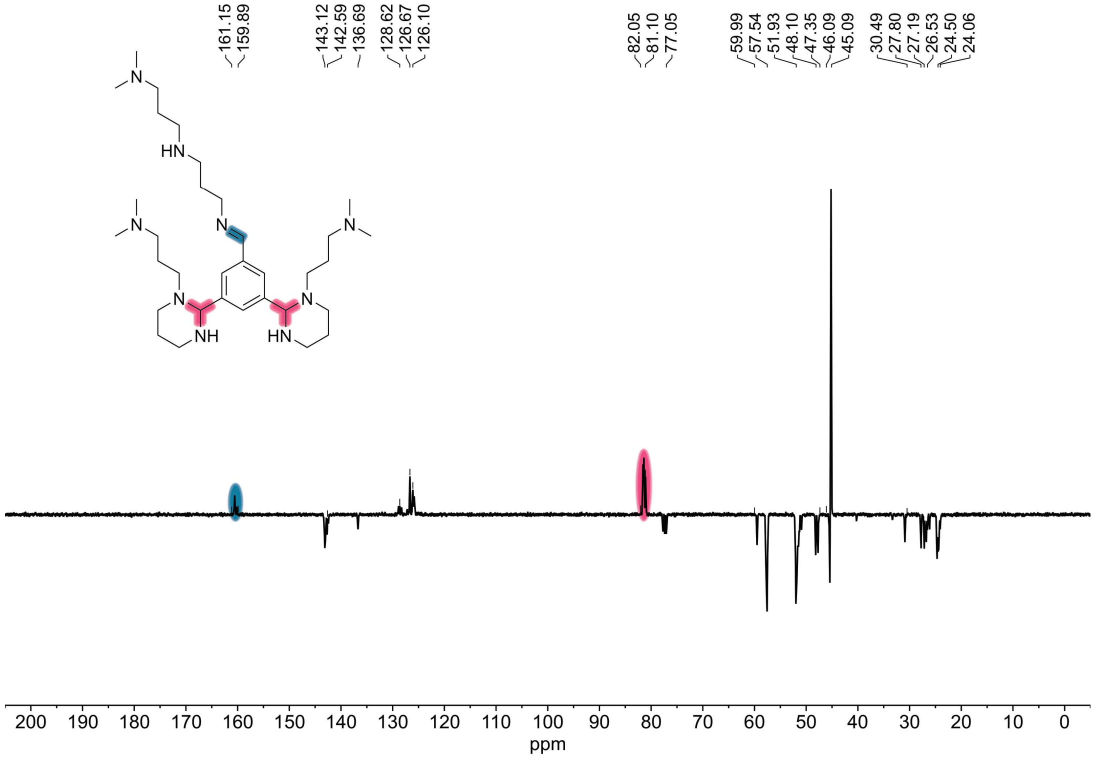

Figure S12. APT spectrum of **A2** in $CDCl_3$.

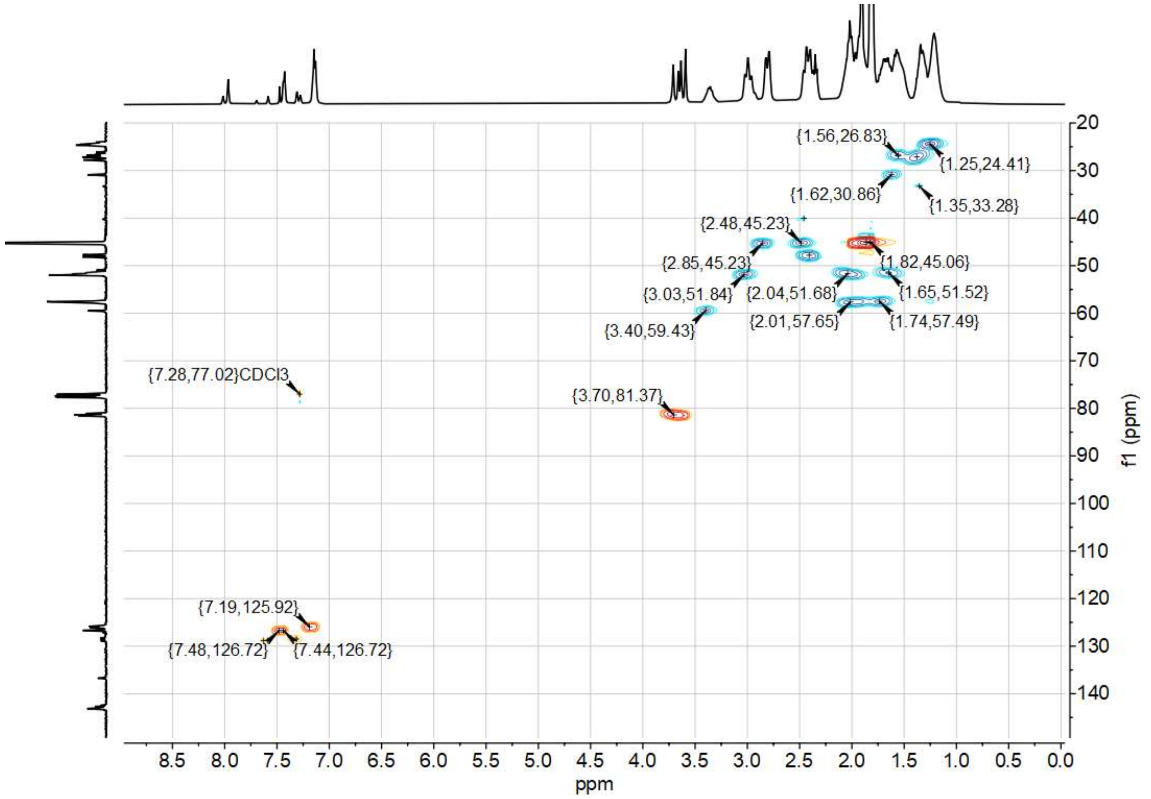

Figure S13. HSQC spectrum of **A2** in $CDCl_3$.

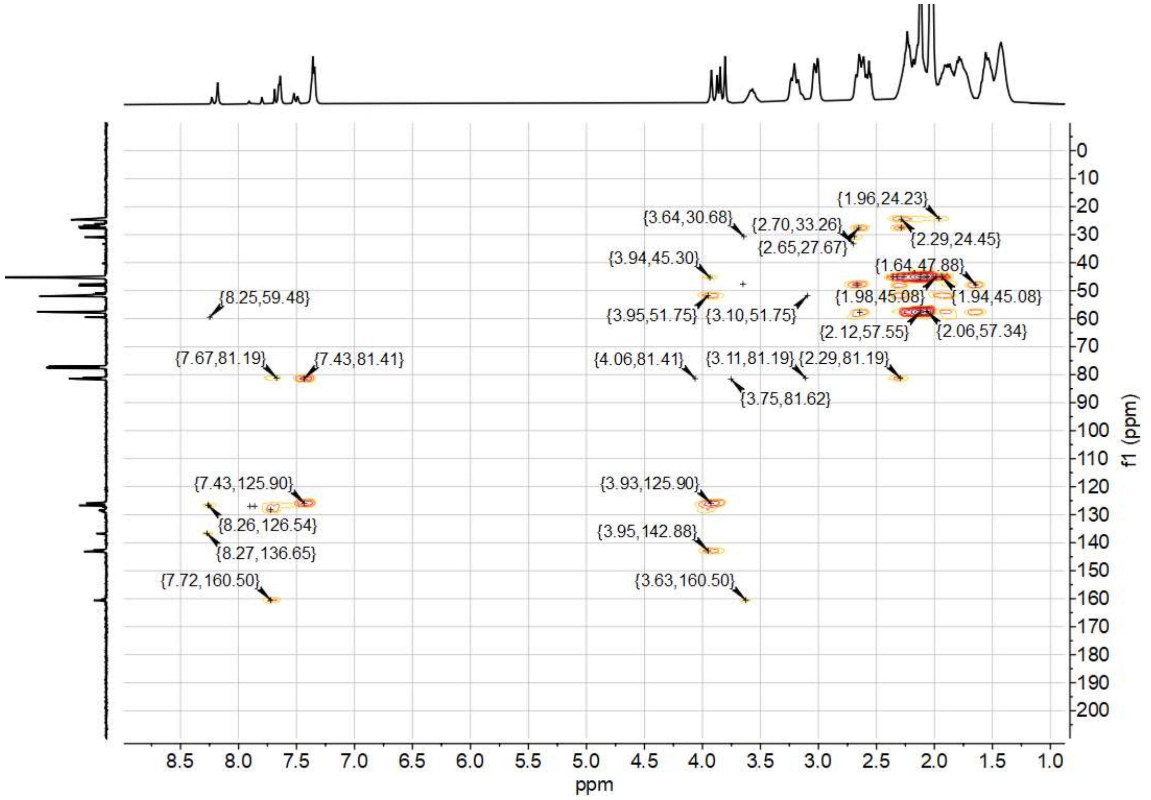

Figure S14. HMBC spectrum of **A2** in $CDCl_3$.

4. Curing Kinetic Studies of P1

The curing kinetics for **P1** were monitored using non-isothermal differential scanning calorimetry (DSC) at heating rates of 5, 10, 15, 20 °C/min. To calculate activation energies of the systems, peak points of curves were utilized with the corresponding heating rates in the Kissinger equation (eqn 1):

$$-\ln\left(\frac{\beta}{T_{p^2}}\right) = \frac{E_a}{RT_{p^2}} - \ln\left(\frac{AR}{E_a}\right) \quad (1)$$

β : the heating rate, T_p: peak temperature, E_a: activation energy, A: pre-exponential factor, R: ideal gas constant.

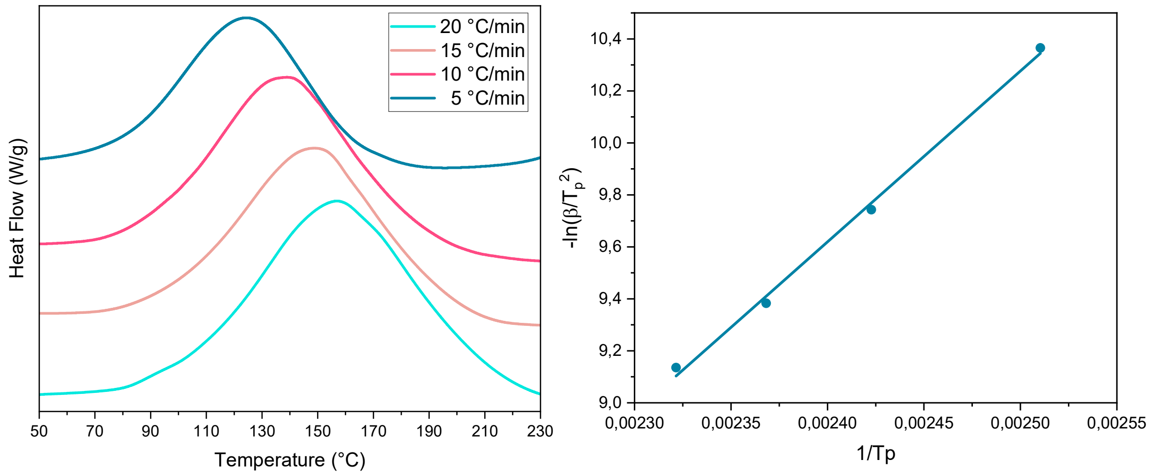

Figure S15. DSC heating curves of **M1** with **DGEBA** at heating rates of 5, 10, 15, and 20 °C/min (left) for **P1**. Linear plot showing $\ln(\beta/T_p^2)$ plotted against $1/T_p$ based on Kissinger's theory.

5. The Curing of the Polymers

5.1 The Curing of **P1**

4 g (6.93 mmol) of **M1** monomer and 1.80 g (10.40 mmol) of **DGEBA** were weighed into a Teflon mold. Then, the monomers were stirred at 40 °C until a homogeneous mixture was obtained. Subsequently, the mixture was placed in a vacuum oven and degassed at 30 °C for approximately 15 minutes. Following degassing, the mixture was transferred to a nitrogen oven pre-set to 150 °C and allowed to cure for 3 hours.

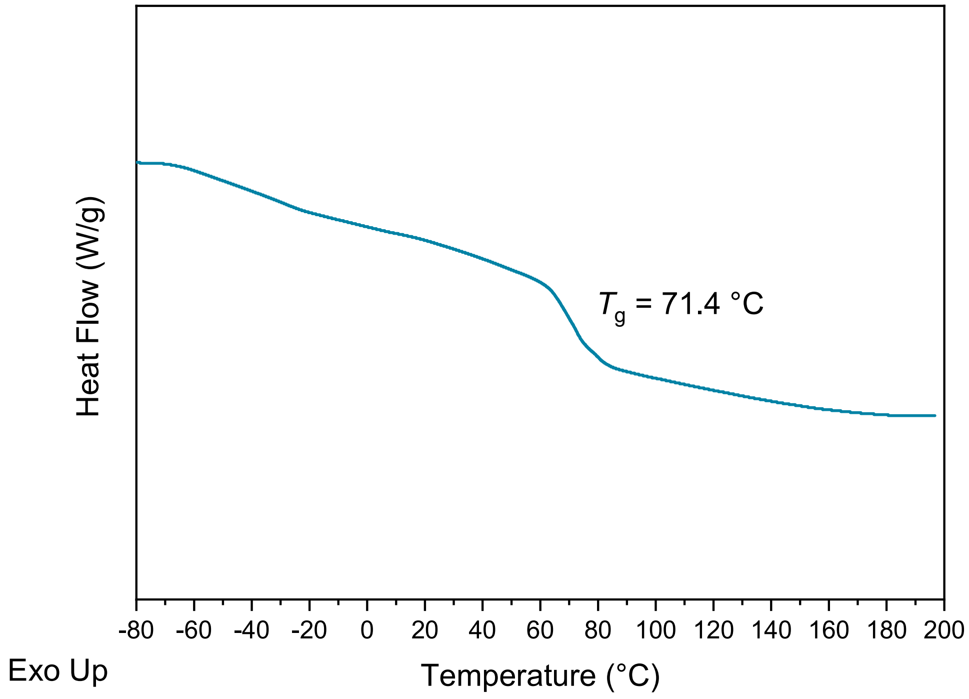

Figure S16. DSC thermogram of **P1** under a N_2 atmosphere with a heating rate of 10 °C/min. (Based on the second heating cycle.)

5.2 The Curing of **P1CF**

2 g (3.47 mmol) of **M1** and 1.77 g (5.20 mmol) of **DGEBA** were weighed into a Teflon mold. The monomers were stirred at 40 °C until a homogeneous mixture was achieved. This mixture was then placed in a vacuum oven and degassed at 30 °C for approximately 15 minutes. Following degassing, 3 plies of carbon fibers (S-CF-22-240-125) were placed into the mold. The mixture was then transferred to a nitrogen oven pre-set to 150 °C and allowed to cure for 3 hours. After curing, the resin was pressed at 120 °C for 1 hour under a pressure of 10 MPa.

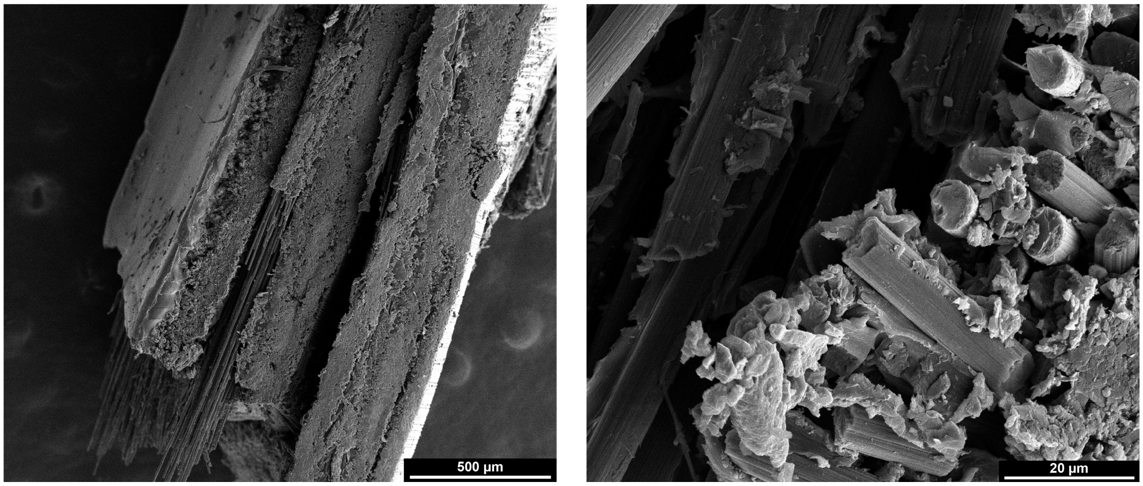

Figure S17. SEM images of shredded tensile test specimen of **P1CF**. The left image displays the specimen at a 500 µm scale, while the right image provides a detailed view at a 20 µm scale.

6. Stress Relaxation Test and Activation Energy Analyses of the Bond Exchange Reaction

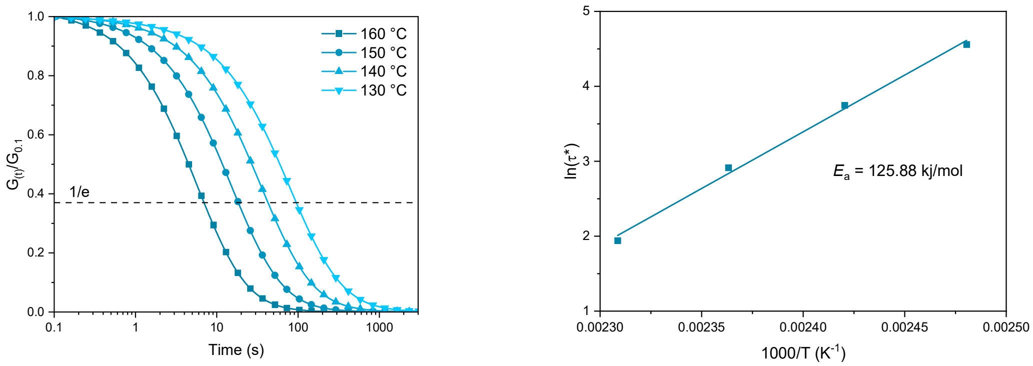

Figure S18. Normalized stress-relaxation of **P1** at different temperatures (130 °C, 140 °C, 150 °C, and 160 °C) (Left) and the fitted curve between 1000/T and the characteristic relaxation time (τ*) according to the Arrhenius law (Right). The activation energy (E_a) of the bond exchange reaction was calculated as 125.88 kJ/mol.

7. Stability Test, Swelling Ratio, and Gel Fraction Calculation

Pieces of **P1** were cut to uniform size. These polymer samples were then sequentially immersed in various solvents: n-hexane, acetone, water, ethanol, methanol, ethyl acetate, acetonitrile, DMSO, and DMF. The samples remained in each solvent for 24 hours, 48 hours, and 72 hours, respectively. After each immersion period, the samples were removed, and any excess solvent on the surface was wiped off. The samples' weights (W_s) were then measured, and the swelling

ratios (q) were calculated by comparing these weights to their initial weights (W_0) using Equation 2:

$$q = 100 \times \frac{W_S - W_0}{W_0} \quad (2)$$

The swelling ratios for each solvent and time point were recorded. Additionally, the gel fractions were determined using Equation 3, where ϕ represents the gel fraction, W_0 is the polymer's initial weight, and W_1 is its weight after drying:

$$\phi = 100 \times \frac{W_1}{W_0} \quad (3)$$

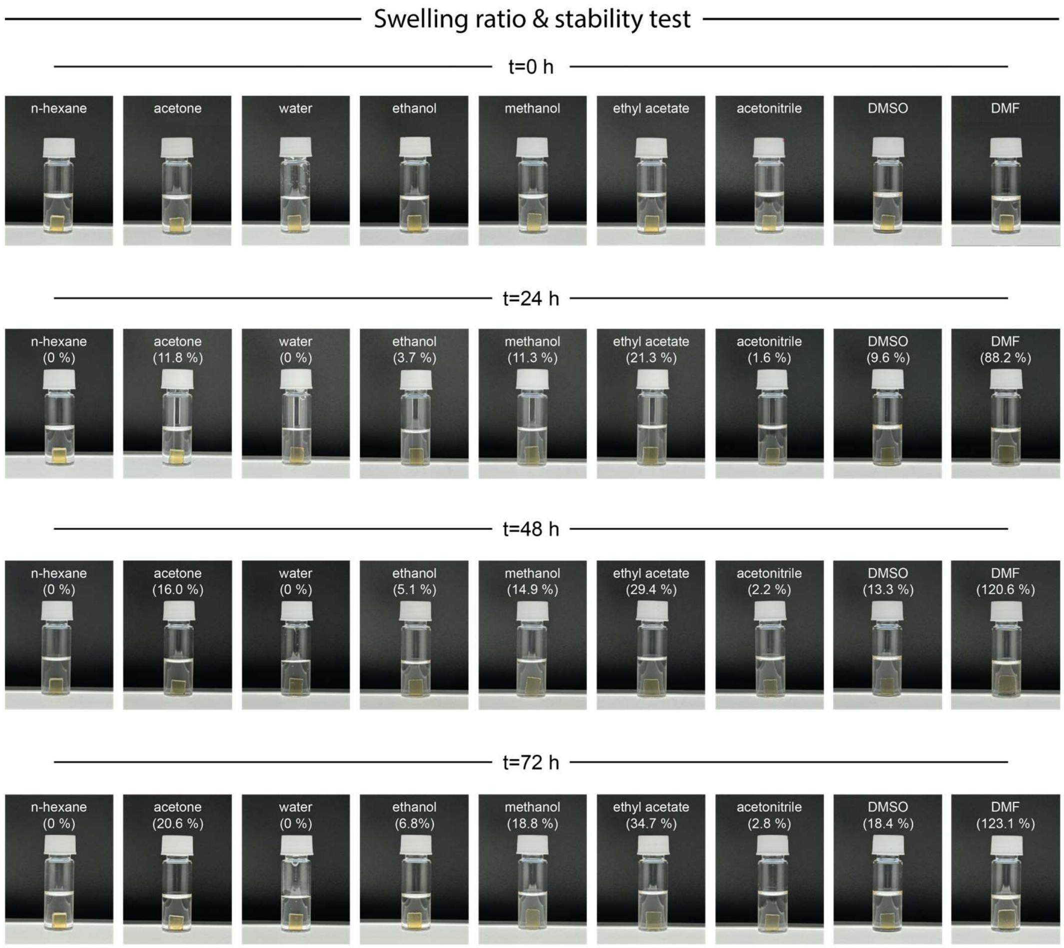

Figure S19. Photographs depicting the stability of epoxy network **P1** in various solvents (n-hexane, acetone, water, ethanol, methanol, ethyl acetate, acetonitrile, DMSO, and DMF; approximately 80 mg of epoxy network in 2 mL of solvent) at time intervals of 0, 24, 48, and 72 hours, alongside the associated swelling ratios for each time point.

8. Depolymerization of P1

In a 100 mL closed-neck container, 4 g of **P1** epoxy thermoset was weighed. Subsequently, 50 mL of 1M HCl solution was added to the container, and the mixture was stirred at room temperature for 16 hours. The resulting heterogeneous solution was filtered through filter paper to obtain solid **1**. The remaining solution was then extracted three times with diethyl ether. The organic fractions were combined, dried over Na_2SO_4, and concentrated to yield pure pale yellow solid **1** (yield = 554 mg, 92.9%). Then the remaining aqueous phase was neutralized with 50 mL of 1M NaOH solution. The resulting heterogeneous solution was then extracted twice with CH_2Cl_2. Organic phases were combined, dried over Na_2SO_4, and concentrated under reduced pressure at 40°C to yield an orange low melting solid **D1** (yield = 3.54 g, 98.3 %). ^{1}H NMR ($CDCl_3$, δ) 7.13-6.80 (m, 8H, C_6H_4), 4.06-3.87 (m, 6H, $PhOCH_2CH$), 2.78-2.52 (m, 18H, NH_2CH_2, NCH_2, NC*H*, & NH_2), 1.80-1.01 (30H, m, CH_3, $CH_2CH_2CH_2$ & CH_2 protons of cyclohexyl). ^{13}C NMR ($CDCl_3$, δ) 156.72, 143.28, 127.69, 113.89, 70.51, 67.06, 59.89, 53.39, 48.64, 41.68, 40.22, 31.80, 31.05, 30.34, 27.58, 26.11. MS (ESI-TOF) *m/z*: $[M+H]^+$ calculated for $C_{39}H_{65}N_4O_4^+$ 653.50, found 653.67.

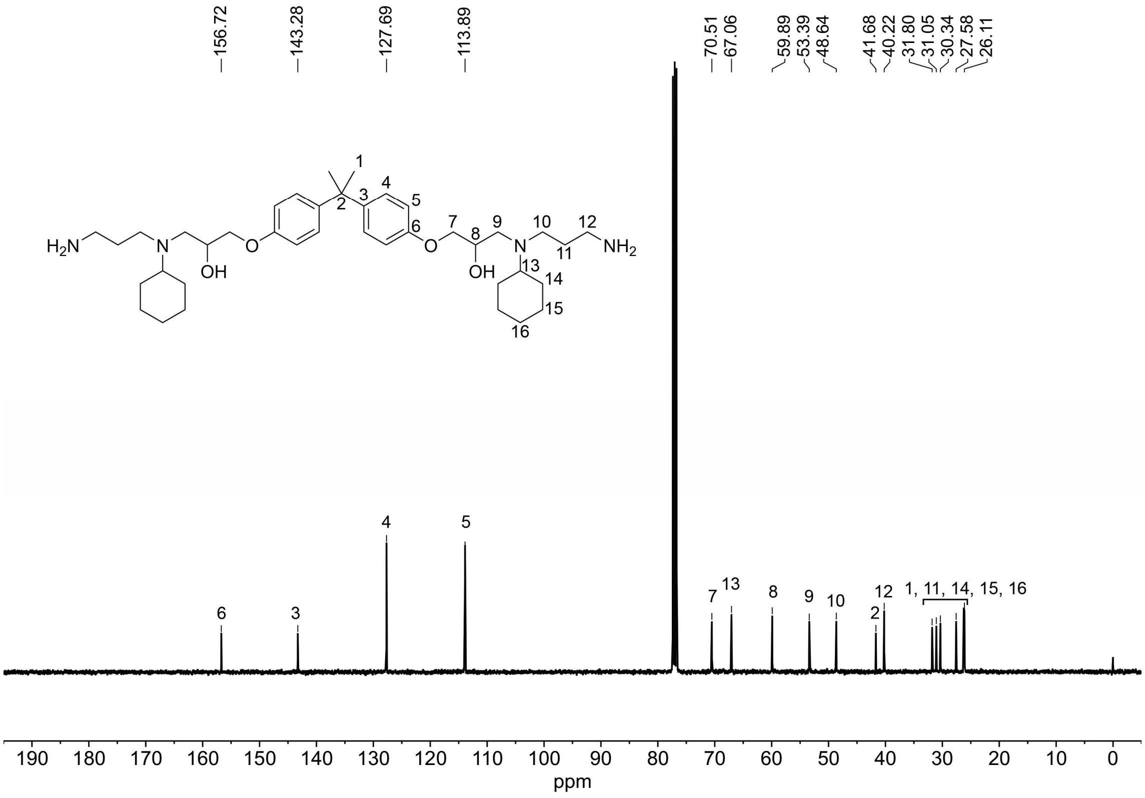

Figure S20. ^{13}C NMR spectrum of **D1** ($CDCl_3$, 101 MHz).

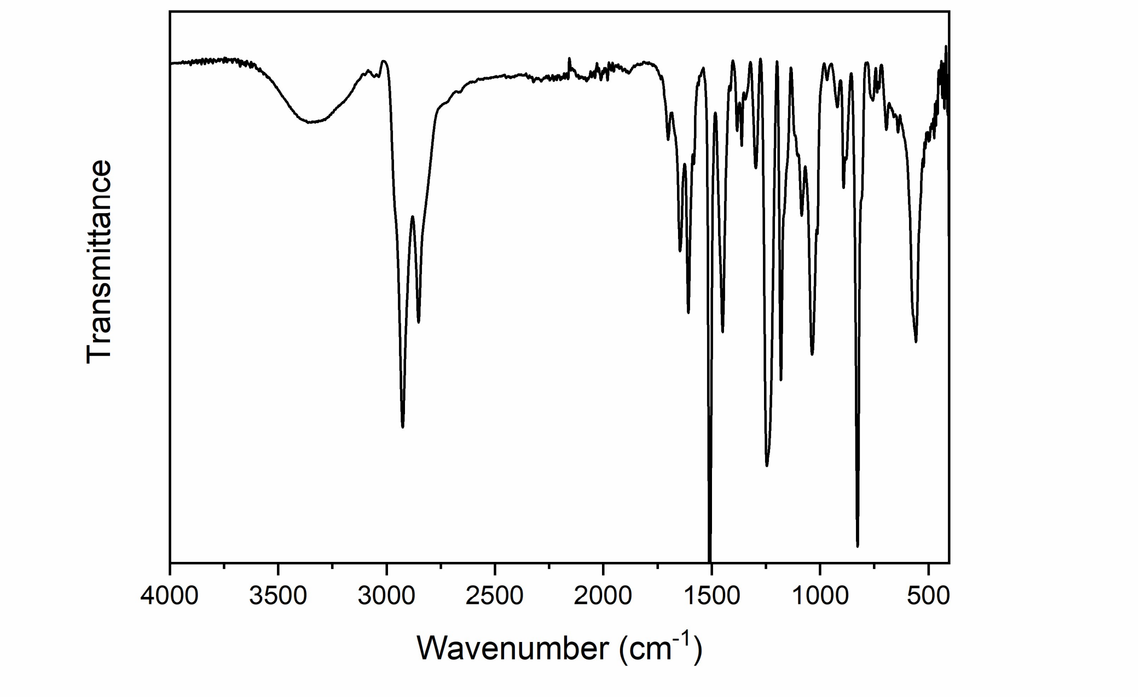

Figure S21. FTIR spectrum of **D1.**

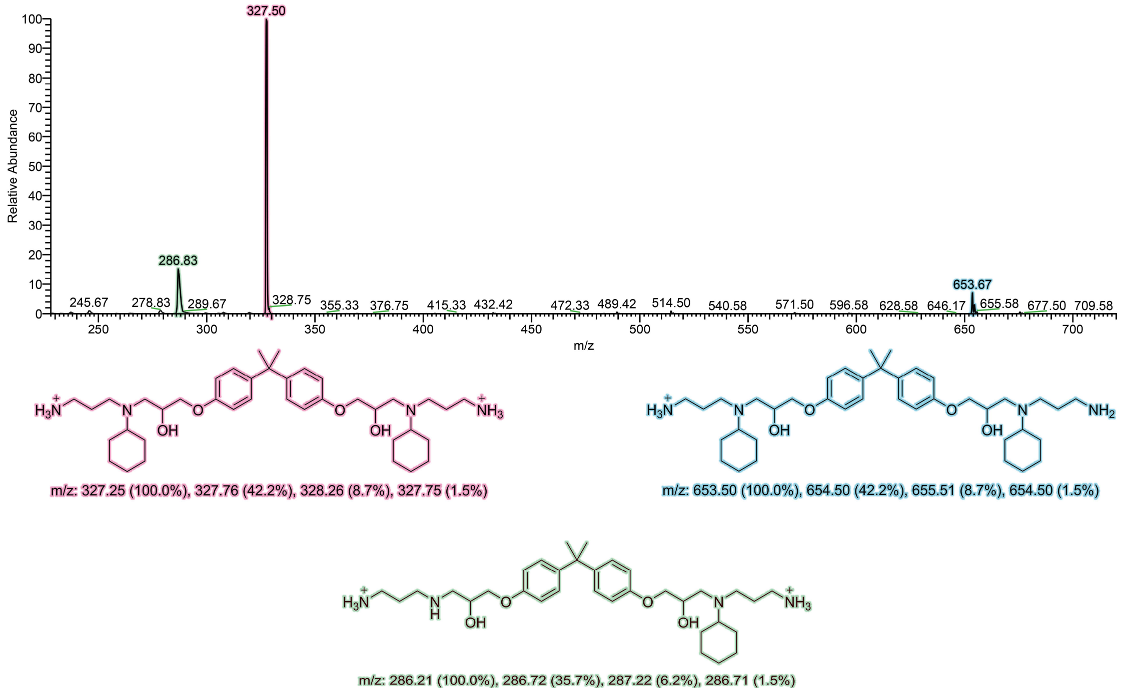

Figure S22. LC-MS spectrum of **D1**.

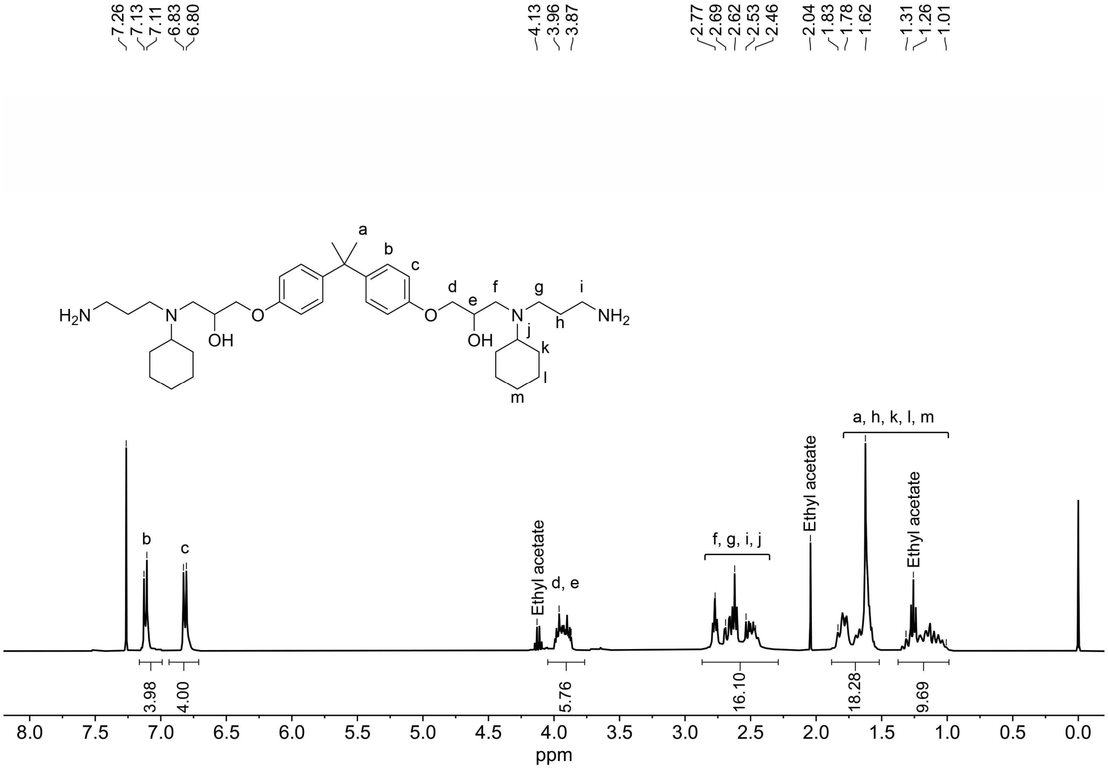

Figure S23. ^{1}H NMR spectrum of **D1** sample recovered from acidic depolymerization of **P1CF** ($CDCl_3$, 400 MHz).

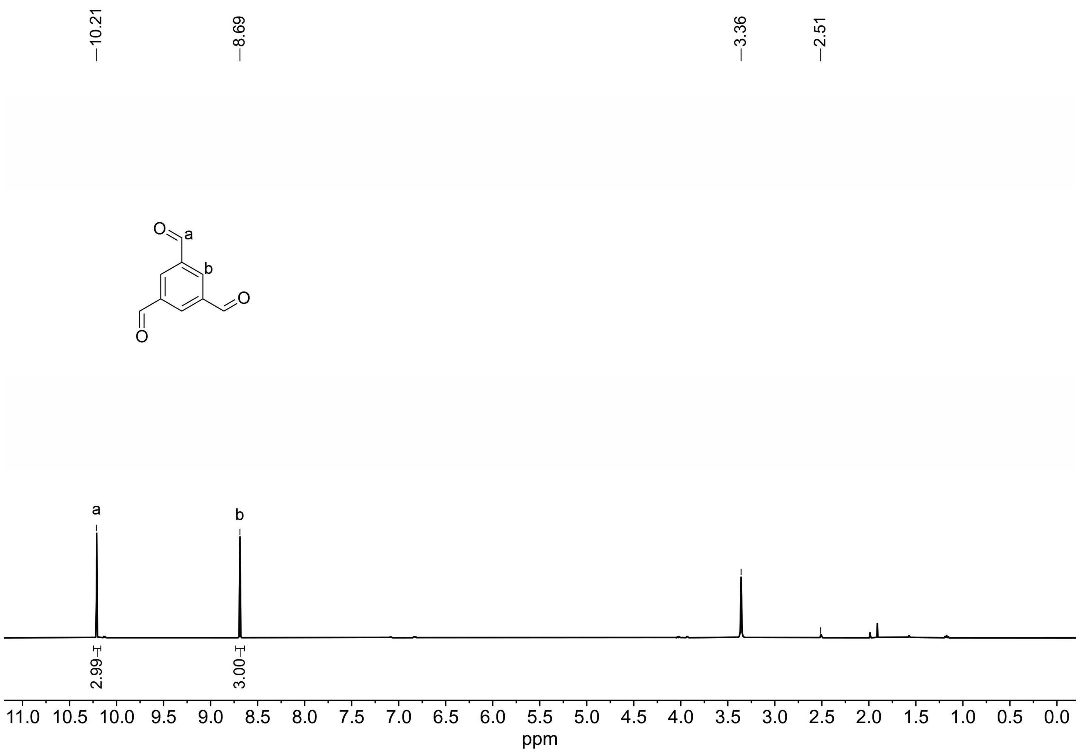

Figure S24. ^{1}H NMR spectrum of **1** sample recovered from acidic depolymerization of **P1CF** ($CDCl_3$, 400 MHz).

9. Closed-Loop Chemical Recycling

3 g of the **D1** recycling product was added to a Teflon mold. Subsequently, 15 mL of THF was added to the mold. To the resulting homogeneous mixture, 431.9 mg of solid **1** was added and quickly stirred at room temperature using a spatula. The mixture gradually became homogeneous, initiating the formation of a thin film. After the evaporation of THF at room temperature, the mold was placed in a nitrogen oven set to 120 °C for 16 hours. The resulting polymer, **P1R**, was then placed into a vacuum oven set to 120 °C overnight to remove any trace amount of THF. Finally, to remove any formed small bubbles, the **P1R** polymer was pressed at 120 °C under 10 MPa pressure for 1 hour.

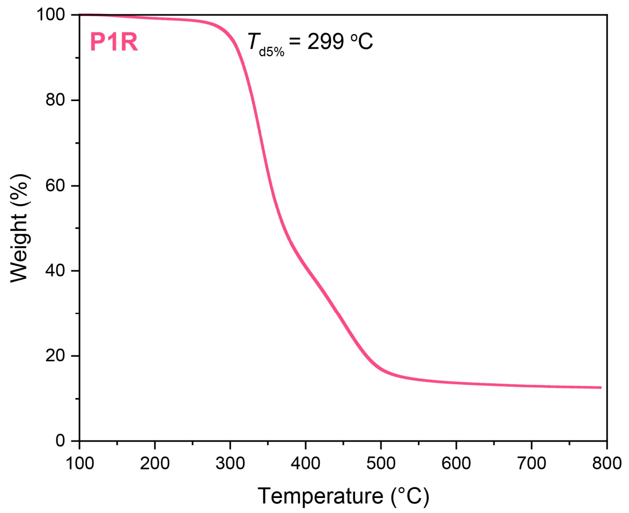

Figure S25. The TGA graph of **P1R** analyzed under a N_2 atmosphere with a heating rate of 10 °C/min from 100 °C to 800 °C.

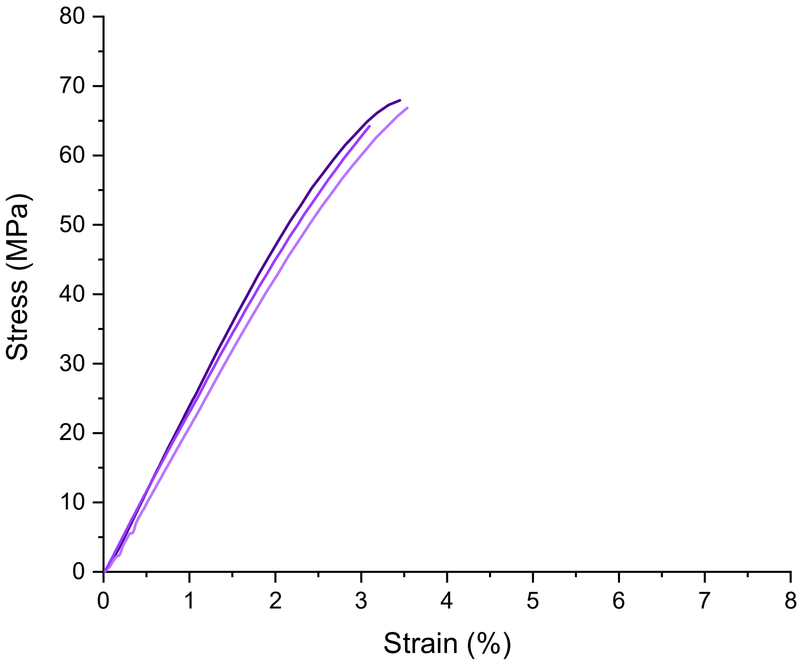

Figure S26. Stress-strain test results of **P1R2**.

10. Solvent-Based Recycling of P1 (P1S)

Approximately 3 g of **P1** was immersed in approximately 50 ml of a CH_2Cl_2. After 3 days of stirring at room temperature, it was observed that the **P1** completely dissolved in CH_2Cl_2. The solution containing **P1** was transferred to a Teflon mold and evaporated at room temperature overnight. After most of the solvent evaporated, the resulting material was placed in a nitrogen oven set to 120 °C for 6 hours. The resulting polymer, **P1S**, was then transferred to a vacuum oven set to 120 °C overnight to remove any remaining THF. Finally, to eliminate any remaining small bubbles, the **P1S** polymer was compressed at 120 °C under 10 MPa pressure for 1 hour.

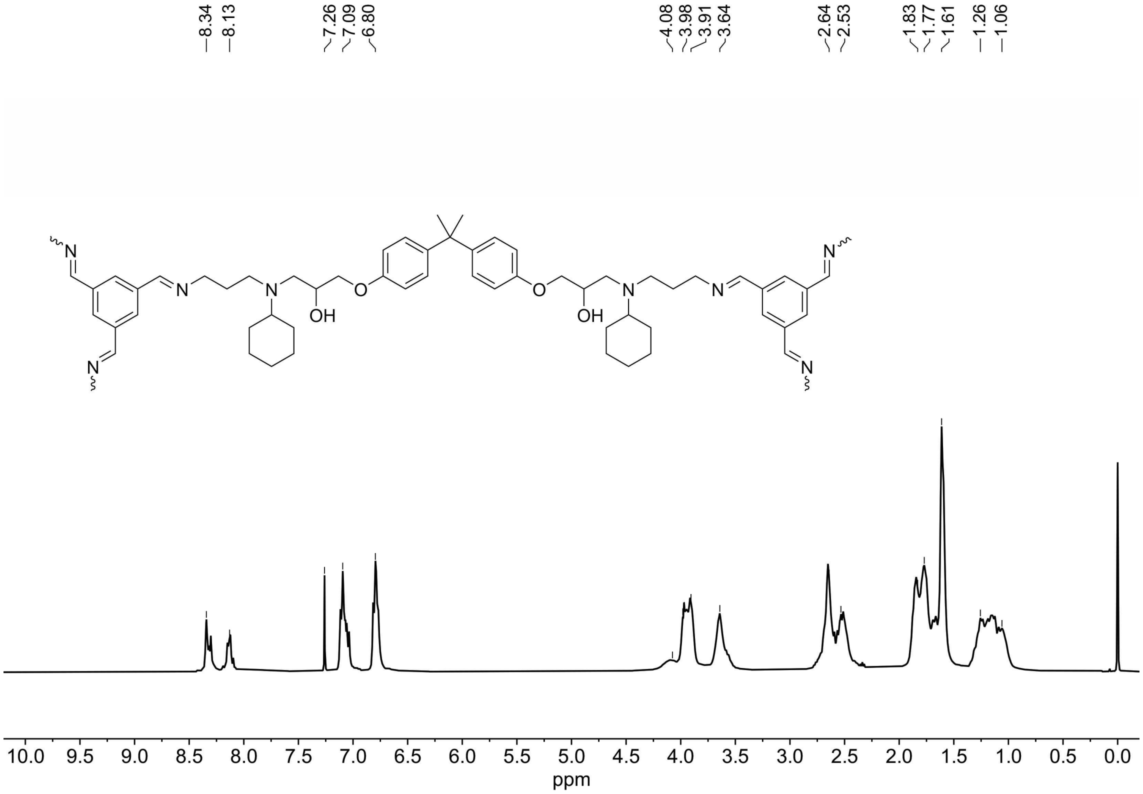

Figure S27. The ^{1}H NMR spectrum obtained from the dissolution of **P1** in $CDCl_3$ (400 MHz).

11. Solvent-Based Recycling of P1CF (P1CFS)

Approximately 2 g of **P1CF** was immersed in 50 ml of CH_2Cl_2. After 3 days of stirring at room temperature, the resin of **P1CF** was observed to have completely dissolved in CH_2Cl_2, while the carbon fibers remained intact. The solution was then filtered and transferred to a Teflon mold, where it was allowed to evaporate at room temperature overnight. After the majority of the solvent had evaporated, the material was placed in a nitrogen oven set to 120 °C for 6 hours. The resulting polymer, **P1CFS**, was subsequently transferred to a vacuum oven set to 120 °C overnight to remove any remaining THF. Finally, to eliminate any residual small bubbles, the **P1CFS** was compressed at 120 °C under a pressure of 10 MPa for 1 hour. The separated carbon fibers were stirred at room temperature for an additional 5 days, with the solvent being renewed daily.

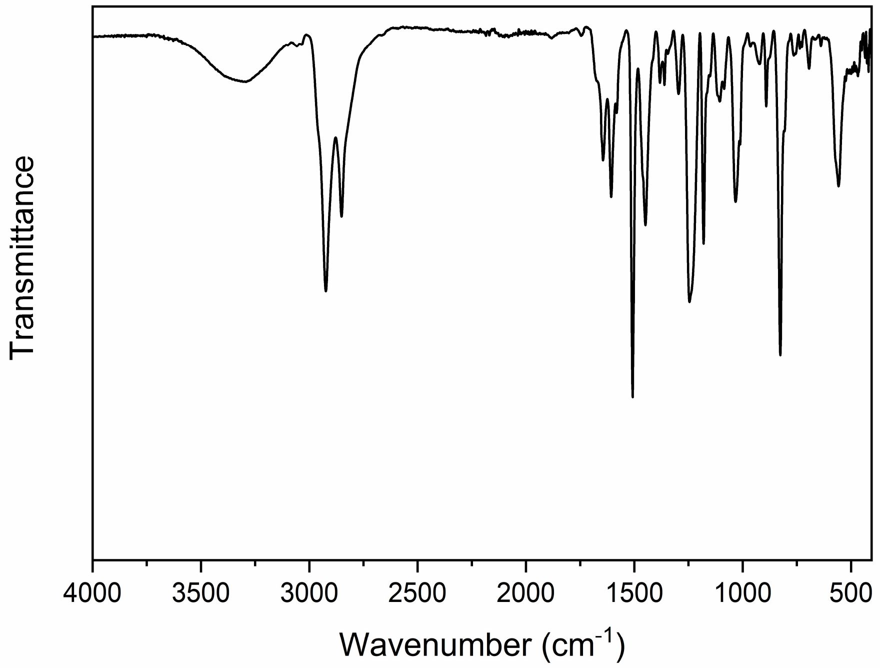

Figure S28. FTIR spectrum of **P1S.**

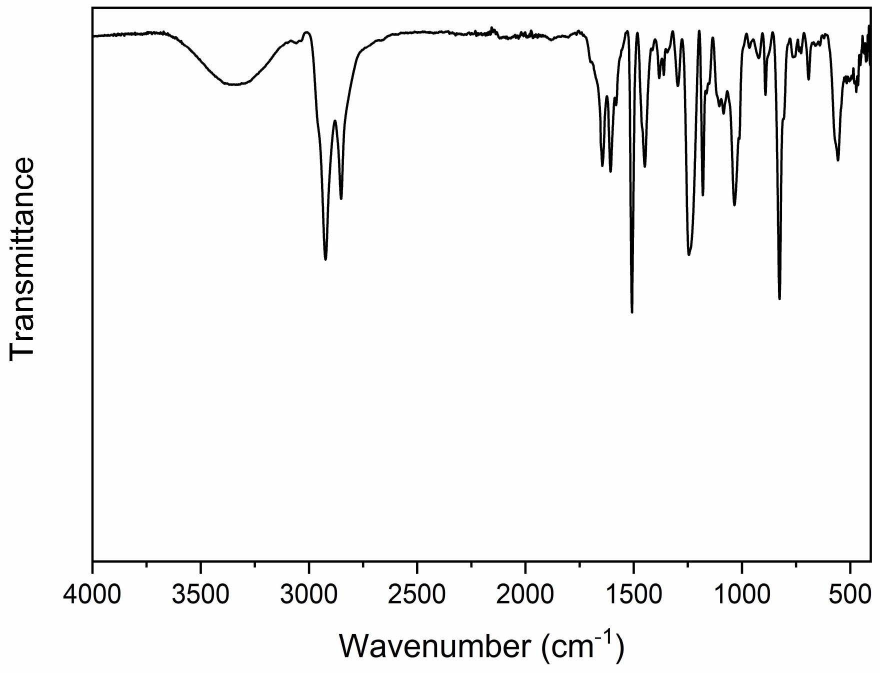

Figure S29. FTIR spectrum of **P1CFS.**

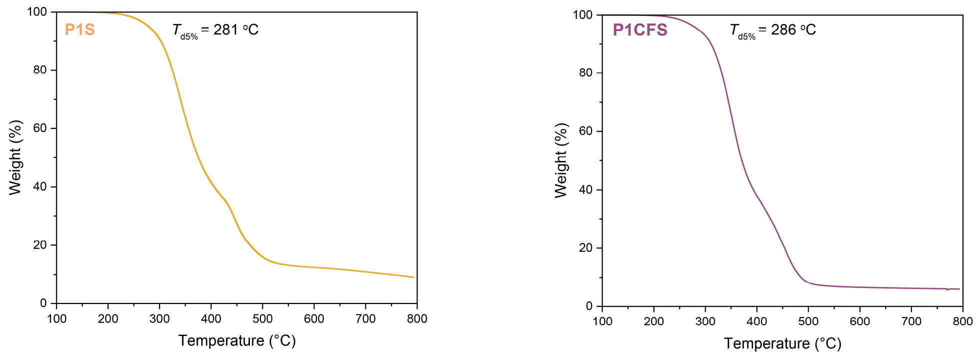

Figure S30. The TGA graphs of **P1S** and **P1CFS** analyzed under a N_2 atmosphere with a heating rate of 10 °C/min from 100 °C to 800 °C.

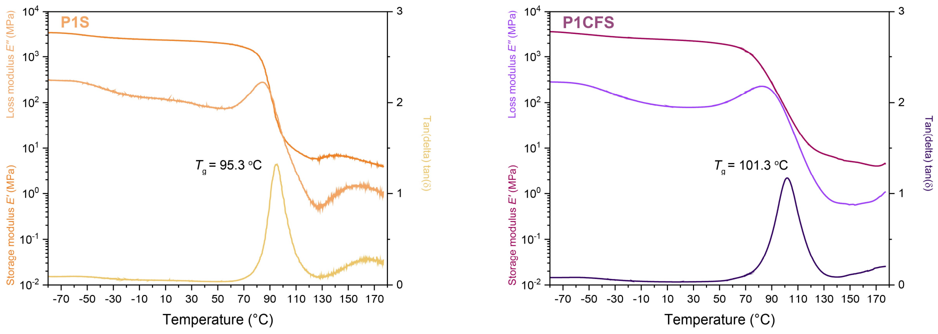

Figure S31. The DMA results of **P1S** and **P1CFS** obtained from a heating rate of 3 °C/min under an oscillatory strain of 0.1% and a frequency of 1 Hz.

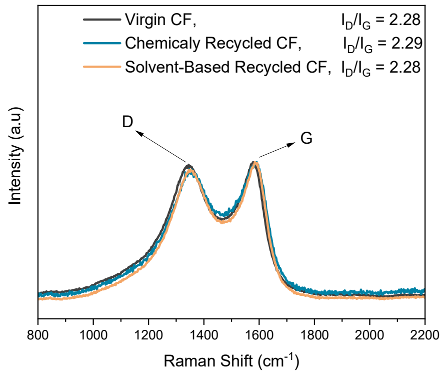

Figure S32. Raman spectra of virgin, chemically recycled, and solvent-based recycled carbon fibers (CF). The D band (~1350 cm^{-1}) and G band (~1580 cm^{-1}) are observed for all samples. The nearly identical I_D/I_G ratios (2.28 for virgin CF, 2.29 for chemically recycled CF, and 2.28 for solvent-based recycled CF) indicate that the structural integrity of the carbon fibers is maintained after recycling.
